# Enzymatic Basis for the Oxidative Branch of Aromatic Amino Acid Fermentation Leading to *p*‐cresol Formation

**DOI:** 10.1002/advs.75061

**Published:** 2026-03-31

**Authors:** Li Jiang, Yifeng Wei, Xumei Liu, Dazhi Liu, Zhenyu Liu, Yang Tong, Jinyu Yin, Ankanahalli N. Nanjaraj Urs, Meining Xing, Mark A. Harrison, Chuyuan Zhang, Sheng Yang, Yunzi Luo, Ee Lui Ang, Huimin Zhao, Yan Zhang

**Affiliations:** ^1^ New Cornerstone Science Laboratory School of Pharmaceutical Science and Technology Tianjin University Tianjin China; ^2^ Tianjin Key Laboratory For Modern Drug Delivery & High‐Efficiency Collaborative Innovation Center of Chemical Science and Engineering School of Pharmaceutical Science and Technology Tianjin University Tianjin China; ^3^ Frontiers Science Center For Synthetic Biology (Ministry of Education) Tianjin University Tianjin China; ^4^ Singapore Institute of Food and Biotechnology Innovation Agency for Science Technology and Research (A^∗^STAR) Singapore; ^5^ Key Laboratory of Systems Bioengineering (Ministry of Education) School of Chemical Engineering and Technology Tianjin University Tianjin China; ^6^ Department of Infection Biology London School of Hygiene and Tropical Medicine London UK; ^7^ Key Laboratory of Synthetic Biology Center For Excellence of Molecular Plant Science Chinese Academy of Sciences Shanghai China; ^8^ Department of Chemical and Biomolecular Engineering University of Illinois at Urbana‐Champaign Urbana Illinois USA; ^9^ School of Life Sciences and Biotechnology Shanghai Jiao Tong University Shanghai China

## Abstract

The phenolic metabolite *p*‐cresol is a byproduct of tyrosine fermentation by certain strictly anaerobic bacteria, including the human gut pathogen *Clostridium difficile*, with toxic effects on the host and intestinal microbiota. The only enzyme in this biochemical pathway characterized to date is the glycyl radical enzyme *p*‐hydroxyphenylacetate (HPA) decarboxylase (HPAD), which catalyzes the terminal step. Here we report the identification and characterization of enzymes for anaerobic degradation of tyrosine to HPA in the model *p‐*cresol‐producing bacterium *Clostridium scatologenes*. In this pathway, tyrosine is first converted to *p‐*hydroxyphenylpyruvate (HPP), followed by net oxidation to HPA by a trio of enzymes HPP: ferredoxin oxidoreductase (HpdDEFG), phosphate HPA‐transferase (HpdJ) and HPA kinase (HpdK). Each step is thermodynamically reversible, and the pathway is coupled to net generation of ATP and reduced ferredoxin. A bioinformatics search reveals that gene clusters containing a similar trio of enzymes are widespread in anaerobic Firmicutes bacteria, suggesting analogous pathways for the degradation of other amino acids. These findings clarify the oxidative pathways by which anaerobic gut bacteria convert aromatic amino acids into a major class of aromatic metabolites including HPA and *p*‐cresol, deepening our understanding of microbiota–host metabolic interactions.

## Graphical abstract

In anaerobic bacteria, tyrosine serves as an electron donor in the oxidative Stickland fermentation branch, generating *p*‐hydroxyphenylacetate, ATP and reduced ferredoxin for decarboxylation into *p*‐cresol.

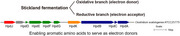

## 1 Introduction

Fermentation of amino acids by strict anaerobic bacteria in the human gut leads to the production of a variety of end‐products, which could be either beneficial or harmful to the host. While most amino acids are converted to beneficial short chain fatty acids, aromatic amino acids are converted to a variety of structurally diverse aromatic metabolites, with potentially harmful effects on the gut microbiota and the host [1, 2, 3, 4, 5]. One such metabolite that has been the subject of extensive research is *p*‐cresol, which is the main end‐product of tyrosine fermentation by the pathogenic bacterium *Clostridium difficile*, a specialist in amino acid fermentation [6]. While most tyrosine‐fermenting Clostridia generate *p‐*hydroxyphenylacetate (HPA) as an end‐product, a small fraction of them, including *C. difficile*, possess the ability to further decarboxylate HPA to form *p‐*cresol.

*C. difficile* is able to tolerate up to 35 mM *p‐*cresol, which is thought to enable active suppression of bacterial competitors under certain growth conditions, leading to dysbiosis of the gut microbiome [6, 7]. In addition, *p*‐cresol and other structurally diverse products of aromatic amino acid fermentation, are absorbed in the intestine and enter the circulatory system, reaching sub‐millimolar concentrations [2]. In humans, gut‐derived *p‐*cresol has been implicated in cardiovascular disease [8] and autism [9, 10]. In the environment, *p‐*cresol is a major component of the “barnyard” odor, and is of great concern to the livestock industry [11]. It is also an oviposition attractant for disease‐spreading *Aedes triseriatus* mosquito [12], and a human‐sweat odorant attracting female *Anopheles* mosquitoes [13]. The chemically challenging HPA decarboxylation reaction is catalyzed by the O_2_‐sensitive glycyl radical enzyme (GRE) *p‐*hydroxyphenylacetate decarboxylase (HPAD), through a mechanism involving free radical chemistry [6]. Another GRE (AAD) was found capable of catalyzing the same reaction but with different radical‐dependent catalytic mechanism [14]. Two other GREs, phenylacetate decarboxylase (PhdB) [15] and indoleacetate decarboxylase (IAD) [16], generate toluene and skatole from the respective products of phenylalanine and tryptophan fermentation. Of the these GRE decarboxylases, HPAD is the most prevalent in existing sequence databases, followed by AAD, IAD and then PhdB [14, 15, 16].

Amino acid‐fermenting Clostridia like *C. difficile* obtain energy through Stickland fermentation, a complex and incompletely explored scheme for energy generation, where certain amino acids serve as electron donors and others as electron acceptors, mediated by reduced ferredoxin (Fdx) as a low potential electron carrier [17]. Aromatic amino acids can serve both as electron donors or acceptors, via the oxidative and reductive branches of the Stickland pathway. In *C. sporogenes*, a model aromatic amino acid fermenting gut bacterium, tyrosine metabolism via the reductive branch involves conversion by an aminotransferase to *p‐*hydroxyphenylpyruvate, followed by net reduction via a series of intermediates to *p‐*hydroxyphenylpropionate [2]. Similar mechanisms convert phenylalanine and tryptophan to phenyl‐ and indole‐propionate respectively. These metabolites are produced by anaerobic gut bacteria and thought to enter the host circulatory system, with systemic effects that are incompletely understood [2]. HPA, the precursor to *p‐*cresol, is a product of tyrosine metabolism via the oxidative branch of the Stickland pathway. Despite longstanding interest, the enzymes and biochemical details involved in this oxidative pathway for tyrosine and other aromatic amino acids remain poorly understood.

The model organism for cresol (and skatole) production is *C. scatologenes*, which is a prolific producer of cresol (and skatole) as a product of tyrosine (and tryptophan) fermentation [18]. Here we report the identification and in vitro biochemical characterization of a pathway, encoded by a gene cluster in *C. scatologenes*, for the conversion of tyrosine to HPA and then to *p‐*cresol, coupled to the formation of ATP and reduced Fdx. We also expand on the findings with a bioinformatics investigation of similar gene clusters in amino acid‐fermenting *Clostridia*.

## 2 Results

### 2.1 A Gene Cluster for Cresol Formation in *C. scatologenes*

**FIGURE 1:**
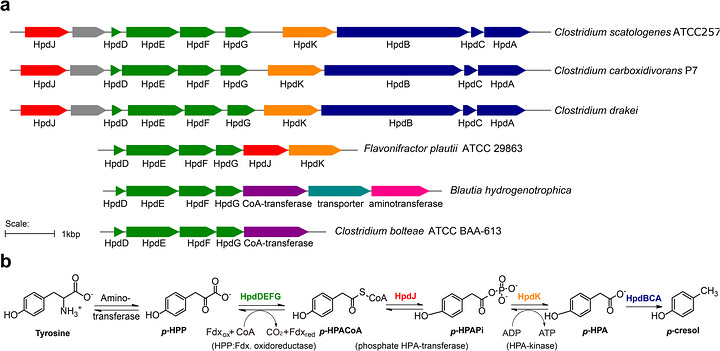
HpdDEFG gene cluster and the proposed tyrosine degradation pathway. a) Genome neighbourhood of close homologs of HpdDEFG in *Clostridium scatologenes* and other bacteria. b) Proposed anaerobic tyrosine degradation pathway in *Clostridium scatologenes*. Fdx_ox_: oxidized ferredoxin, Fdx_red_: reduced ferredoxin. HPAD: encoded by *HpdBCA*.

The first step of the tyrosine fermentation pathway is thought to involve conversion of tyrosine to hydroxyphenylpyruvate (HPP), catalyzed by a pyridoxal 5'‐phosphate (PLP)‐dependent tyrosine aminotransferase, a homolog of which was previously identified in the genome sequence of *C*. scatologenes [19]. To find other candidate genes involved in the production of HPA as a substrate of HPAD, we examined the genome neighborhood of HPAD in diverse bacteria. The genes in the HPAD neighborhood in *C. scatologenes* (ENA genome ID: CP009933) form a gene cluster encoding a possible pathway for the conversion of HPP to HPA (Figure 1a). Identical gene clusters are also present in *C. drakei* and *C. carboxidivorans* P7 (ENA: CP020953 and ACVI01000037 respectively), which are closely related to *C. scatologenes* [18, 20] (Figure 1a), but not in other HPAD‐containing bacteria including *C. difficile*.

The *C. scatologenes* HPAD gene cluster contains a homolog of pyruvate:ferredoxin oxidoreductase (PFOR), containing four subunits, which we named HpdDEFG. We hypothesized that this enzyme oxidizes HPP to HPA‐CoA, concomitant with the reduction of Fdx (Figure 1b). We examined the genome neighborhood of HpdE homologs in the UniRef cluster UniRef50_A0A174NPZ1 (where each member shares ≥ 50% sequence identity and ≥ 80% overlap with the seed sequence of the cluster [21]) and observed the occurrence of HpdDEFG in different organisms (Figure 1a), suggesting that the four genes form a functional unit.

The gene cluster also contains a homolog of phosphate butyryltransferase and a homolog of butyrate kinase, which we named HpdJ and HpdK respectively. We hypothesized that HpdJ and HpdK together catalyze the hydrolysis of the HPA‐CoA, coupled to the phosphorylation of ADP to form ATP (Figure 1b). The proposed pathway is analogous to the pathway for oxidation of alanine and branched amino acids in Stickland fermentation [17]. To further investigate this hypothesis, we produced recombinant proteins and carried out detailed biochemical characterization.

### 2.2 Characterization of HpdDEFG, a HPP‐Fdx Oxidoreductase

An analysis of the domain structure of homologs of PFOR is given by Yan et al. [22]. These proteins typically exhibit the same overall 3D structure exemplified by *Desulfovibrio africanus* PFOR (*Da*PFOR, PDB accession 1B0P [23]), which is an α_2_ homodimer. In other PFOR homologs, protein domains are split into multiple ORFs, leading to variations in the subunit composition [24]. Superposition of the individual HpdDEFG subunit AlphaFold structures with the *Da*PFOR complex as a template suggests that the enzyme is a (HpdDEFG)_2_ octamer, which was supported by modeling of the complex using AlphaFold3 [25] (Figure S1). HpdF contains three conserved Cys for binding of the catalytic [4Fe‐4S] cluster. HpdE and HpdG are predicted to share a tight interface, and the catalytic thiamine pyrophosphate (TPP) cofactor binds at the interface of HpdEG and HpdF. HpdD corresponds to the Fdx domain, containing eight conserved Cys binding two [4Fe‐4S] clusters, putatively involved in electron transfer from the catalytic [4Fe‐4S] cluster.

The genes encoding the HpdEFG subunits were cloned into a pET28a plasmid with individual ribosome binding sites for each gene, and an N‐terminal His_6_‐tagged HpdG followed by HpdE and HpdF. SDS‐PAGE analysis showed that all three subunits were robustly and solubly co‐expressed in roughly equal proportions in *E. coli* BL21 (DE3) cells. The TALON affinity column pulled down HpdG and HpdE in a roughly 1:1 ratio, but only a substoichiometric amount of HpdF (Figure S2a), suggesting a weak interaction between HpdF with the other two subunits. This result is consistent with the model of the (HpdDEFG)_2_ complex, which predicts a tighter interaction between HpdE and HpdG (Figure S1). HpdF thus had to be expressed and purified separately (Figure S2b), followed by cleavage of the N‐terminal His_6_‐tag with TEV protease to avoid interference with Fe‐S cluster reconstitution. All three proteins were degassed on a Schlenk line and mixed in an approximately 1:1:1 ratio in the glove box, followed by reconstitution of the [4Fe‐4S] cluster (Figure S2c). To increase its solubility, HpdD was expressed with an N‐terminal MBP fusion. After purification (Figure S2d), MBP‐HpdD was degassed and brought into the glove for reconstitution of Fe‐S clusters, followed by cleavage of the N‐terminal MBP with TEV protease.

**FIGURE 2:**
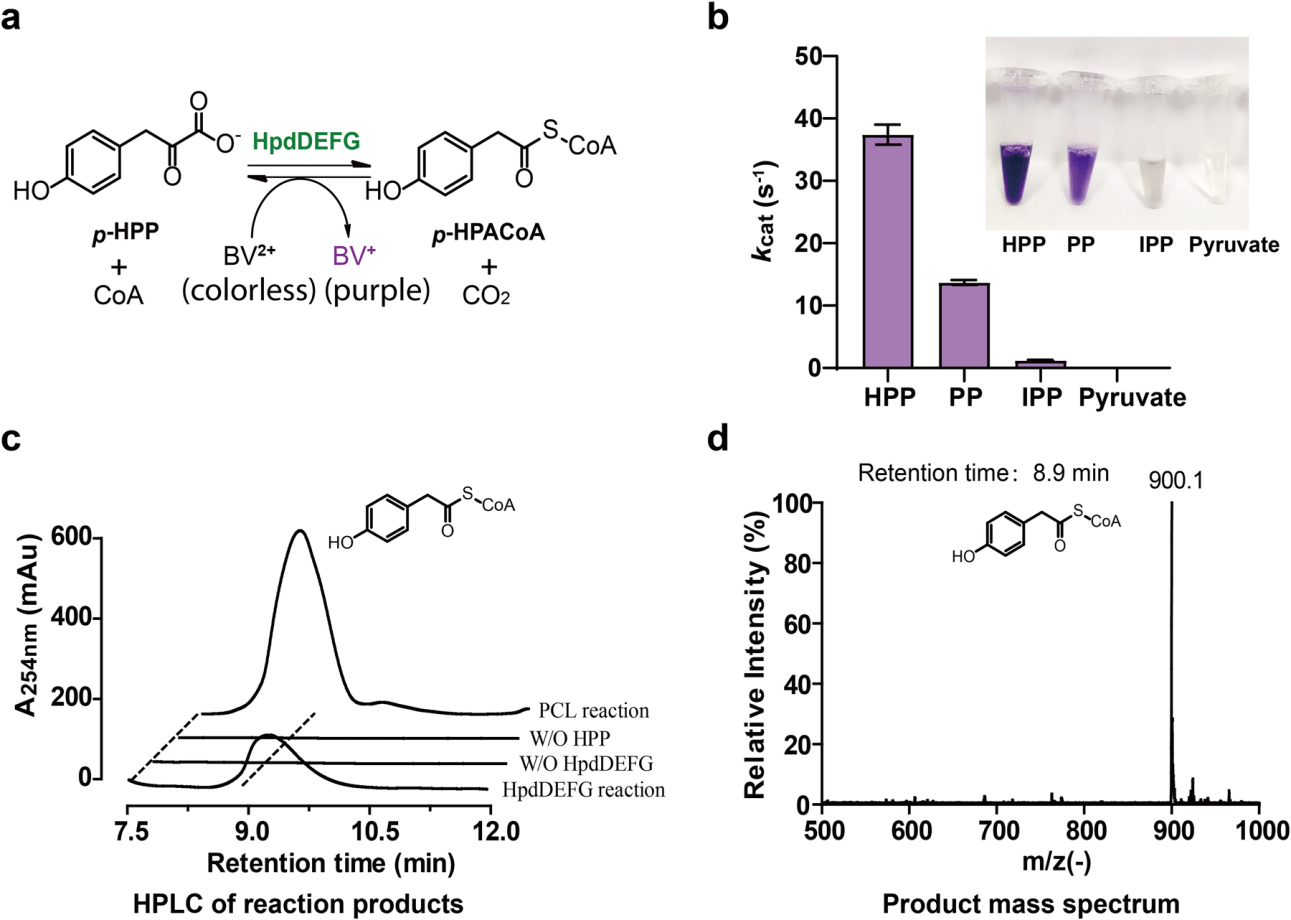
Activity assays for HPP‐Fdx oxidoreductase. a) A scheme of the HpdDEFG catalytic reaction. b) Assays monitoring HpdDEFG‐catalyzed reduction of BV^++^ to BV^+^ with different substrates: HPP, PP, IPP, Pyruvate. c) Elution profiles of the reaction products, monitoring absorbance at 254 nm. Included are: complete assay; negative controls omitting HpdDEFG enzyme or HPP substrate; and HPA‐CoA standard synthesized enzymatically with PCL. d) ESI (‐) m/z spectrum of the HPA‐CoA peak in c.

Reconstituted HpdEFG was mixed with reconstituted HpdD in a 1:1 ratio, and assayed in the presence of HPP and CoA (Figure 2a). Benzyl viologen (BV^2+^) was used as the electron acceptor following a previously reported procedure for 2‐oxoglutarate:Fdx oxidoreductase [26, 27], which allows a colorimetric readout of enzyme activity through the formation of the blue‐purple BV^+^ (Figure 2b). The reaction was also analyzed by LC‐MS, and compared to a HPA‐CoA standard synthesized enzymatically using phenylacetyl‐CoA (PA‐CoA) ligase (PCL) [28]. The HPLC elution profile (Figure 2c) and mass spectrum of the product (Figure 2d) demonstrate the formation of HPA‐CoA. No reaction was observed in negative controls omitting the enzyme HpdDEFG or substrate HPP. Compared to HPP, HpdDEFG exhibited a lower activity with PP (phenylpyruvate) and IPP (indolepyruvate) as substrates, and was inactive with pyruvate as a substrate (Figure 2b). Its kinetic parameters were measured (Figure S3), and summarized in Table 1.

**TABLE 1:** Kinetic parameters of HpdDEFG.

Reaction substrates	*k_cat_* (s^−1^)	*K*_m_ (µm)	*k_cat_* /*K*_m_ (m·s^−1^)
HPP	37.4 ± 1.6	6.0 ± 0.9	6.2 × 10^6^ ± 9.7 × 10^5^
PP	13.7 ± 0.4	17.9 ± 2.5	7.7 × 10^5^ ± 1.1 × 10^5^
IP	1.2 ± 0.1	766.4 ± 167.6	1.6 × 10^3^ ± 3.7 × 10^2^

### 2.3 Characterization of HpdJ, a Phosphate HPA‐transferase

Purified recombinant HpdJ (Figure S4) was assayed in the presence of phosphate and the substrate HPA‐CoA, synthesized enzymatically using PCL. Release of the CoASH sulfyhydryl group, corresponding to phosphotransfer from PA‐CoA to HPA, was monitored spectrophotometrically using DTNB (5,5‐dithio‐bis‐(2‐nitrobenzoic acid), Ellman's reagent), following a previously reported procedure for phosphate acetyltransferase [29]. Time‐ and enzyme dose‐dependent CoASH release was observed (Figure S5), and the kinetic parameters were measured (Figure S6), and summarized in Table 2. Omission of HpdJ or phosphate, abolished the reaction. HpdJ also displayed significant activity with PA‐CoA as a substrate (Figures S5b and S6b; Table 2). The assays demonstrate that HpdJ is a phosphate HPA‐transferase.

**TABLE 2:** Kinetic parameters of HpdJ.

Reaction substrates	*k_cat_* (s^−1^)	*K*_m_ (µm)	*k_cat_* /*K*_m_ (m·s^−1^)
HPA‐CoA	64.1 ± 4.1	8.7 ± 1.7	7.4 × 10^6^ ± 1.5 × 10^6^
PA‐CoA	118.4 ± 8.9	24.2 ± 4.7	4.9 × 10^6^ ± 1.0 × 10^6^

### 2.4 Characterization of HpdK, a HPA‐Kinase

Purified recombinant HpdK (Figure S4) was assayed in the presence of ATP and HPA, and the ADP produced was detected using a pyruvate kinase / lactate dehydrogenase continuous coupled spectrophotometric assay. Time‐ and enzyme dose‐dependent decrease in the A_340_ was observed, indicating the production of ADP (Figure S7). The kinetic parameters were measured (Figure S8), and summarized in Table 3. A much lower activity was detected with PA (Figures S7b and S8b), and IA (indoleacetate) (Figures S7c and S8c) as substrates. The assays demonstrate that HpdK is a HPA kinase.

**TABLE 3:** Kinetic parameters of HpdK (reverse reaction).

Reaction substrates	*k_cat_* (s^−1^)	*K*_m_ (mm)	*k_cat_* /*K*_m_ (m·s^−1^)
HPA	115.9 ± 5.8	3.4 ± 0.9	3.4 × 10^4^ ± 9.2 × 10^3^
PA	2.2 ± 0.1	9.7 ± 1.9	2.3 × 10^2^ ± 4.6 × 10^1^
IA	0.05 ± 0.003	4.2 ± 0.9	1.1 × 10^1^ ± 2.4

### 2.5 In Vitro Reconstitution of the Pathway Converting HPP to *p*‐cresol

**FIGURE 3:**
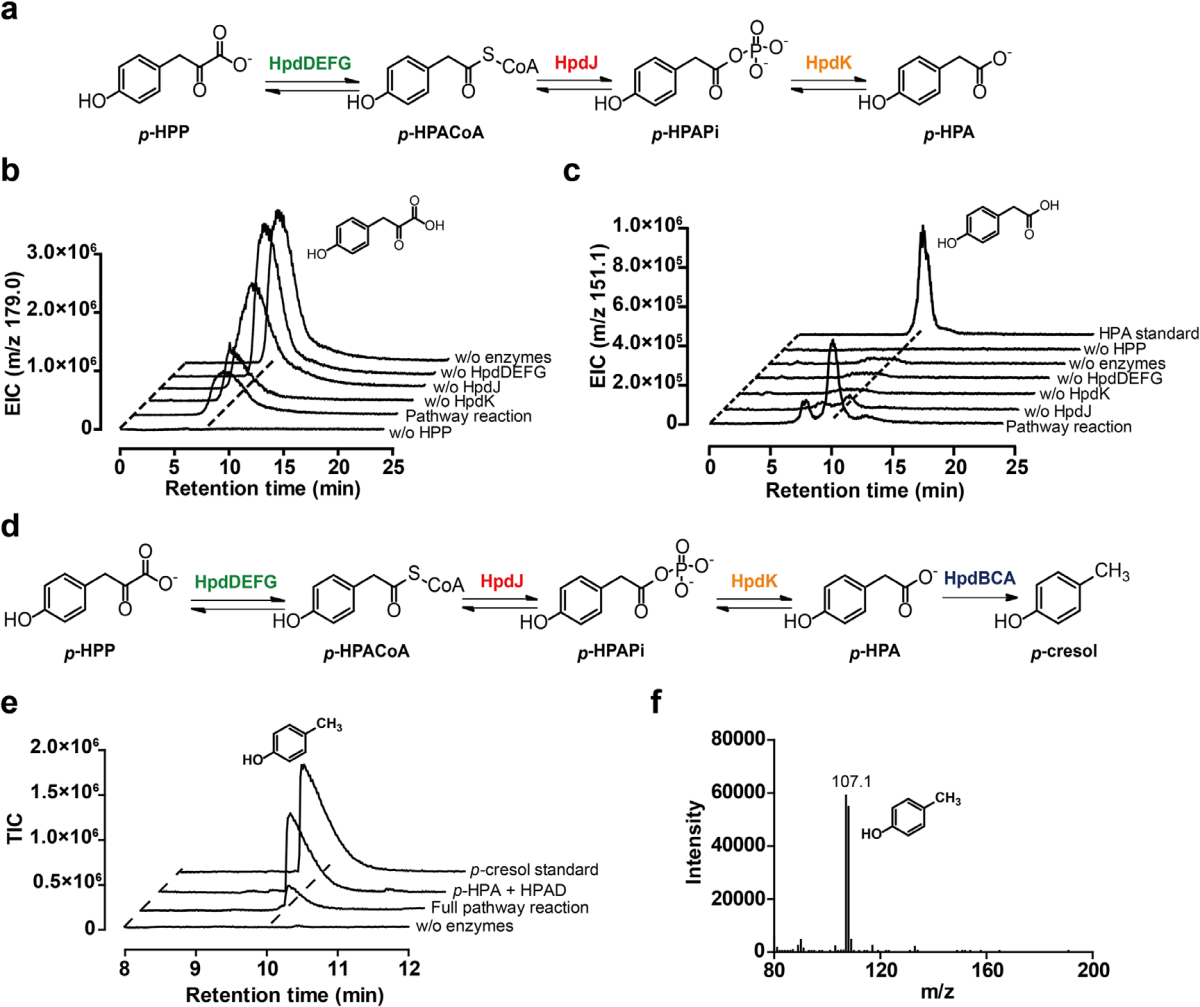
In vitro reconstitution of the tyrosine degradation pathway. a) The schematic diagram depicts the conversion of HPP to HPA by *C. scatologene*s. b) Extracted ion chromatographs monitoring the mass of HPP. c) Extracted ion chromatographs monitoring the mass of HPA. d) The schematic diagram depicts the conversion of HPP to *p*‐cresol by *C. scatologenes*. e) TIC of *p*‐cresol. TIC: total ion chromatogram. f) Mass spectrum of the *p*‐cresol product peak in c.

To test the viability of the proposed pathway, HPP, TPP, CoASH, ADP, and phosphate were incubated in the presence of benzyl viologen and equal amounts (10 µm) of HpdDEFG, HpdJ, HpdK (Figure 3a). The conversion of HPP into HPA was detected by LC‐MS (Figure 3b,c), which was accompanied by the reduction of benzyl viologen. The extracted ion chromatographs monitoring the mass of HPP (Figure 3b) and HPA (Figure 3c), together with the negative ionization mass spectra (Figure S9) shows that HPA was formed in the complete pathway reaction and was eluted at 10.0 min, consistent with the HPA molecular standard in the extracted ion chromatogram (Figure 3c). Negative controls omitting HpdDEFG, HpdK, all enzymes or HPP do not produce HPA (Figure 3c). The assay omitting HpdJ showed trace amount of HPA (Figure 3c), of unknown origin. One possible source of HPA is hydrolysis of the HPA‐TPP intermediate of HpdDEFG upon prolonged incubation, although further mechanistic investigations are needed. The experiment was also conducted with the inclusion of activated HPAD (Figure 3d). Formation of *p*‐cresol was detected by GC‐MS in the positive control containing HPAD with HPA as the substrate, and in the full assay containing HpdDEFG, HpdJ, HpdK and HPAD with HPP as the starting substrate (Figure 3e,f), demonstrating the ability of these enzymes to convert HPP to *p‐*cresol.

### 2.6 Growth of *C. scatologenes* in Tyrosine‐Containing Media

**FIGURE 4:**
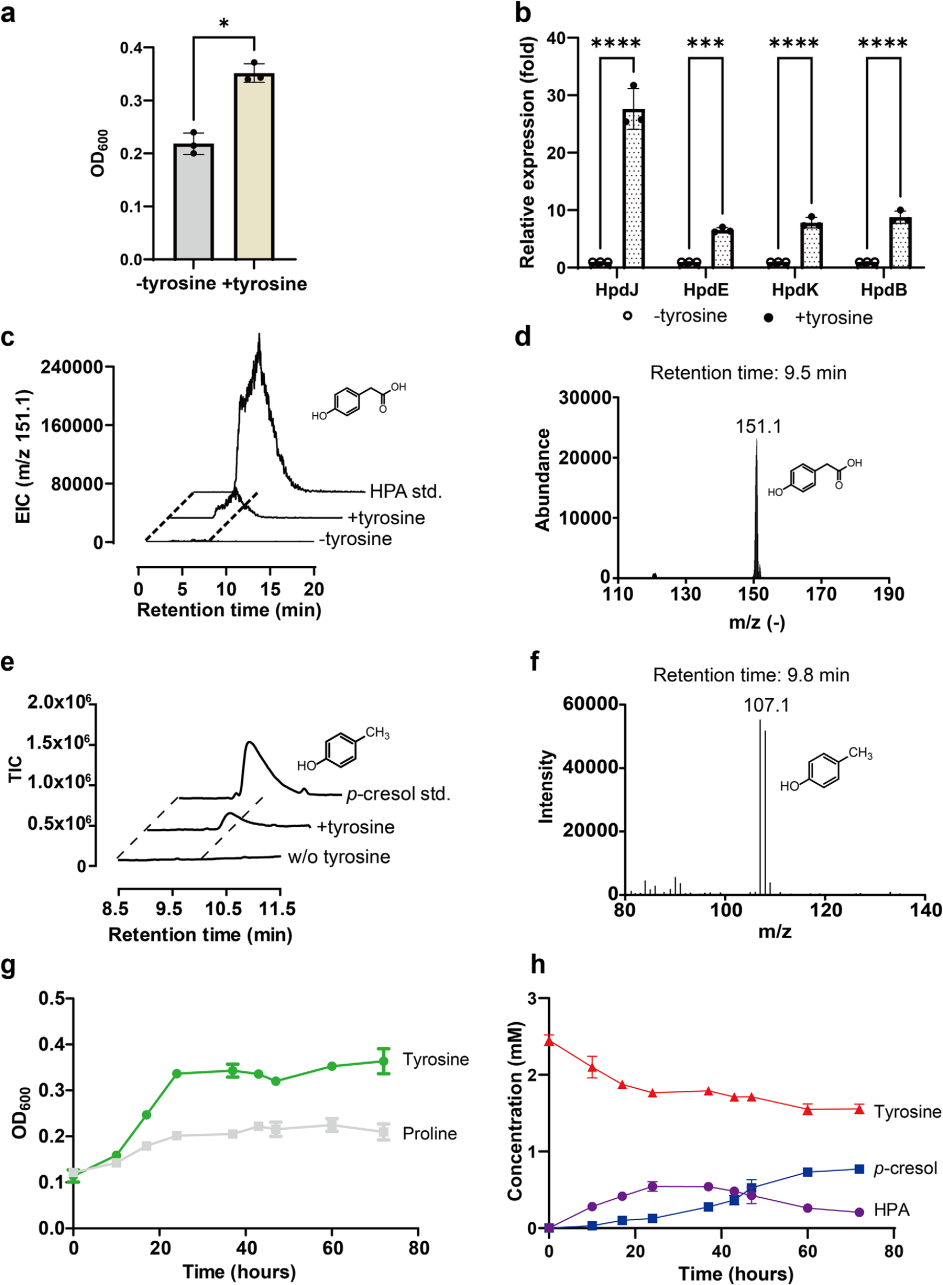
Growth assays of *C. scatologenes*. a) The growth of *C. scatologenes* with or without tyrosine. ^*^ indicates a statistically significant difference (*p* < 0.05, determined by Student's *t*‑test). b) qPCR analyses of the transcription levels of pathway enzymes. The transcriptional levels of genes of interest were normalized by that of the 16S rRNA. Error bars represent the standard deviation values. Significance was determined by two‐way ANONA, ^∗∗∗∗^ *p* < 0.0001, ^∗∗∗^ *p* < 0.001. c) Extracted ion chromatographs monitoring the mass of HPA. d) ESI (‐) m/z spectrum of the HPA peak in c. e) GC‐MS elution profiles monitoring total ion chromatogram (TIC). f) Mass spectrum of the product *p*‐cresol. g) Optical density of *C. scatologenes* cultures grown with tyrosine (green) or proline (grey) was monitored over time. h) The concentration of tyrosine ([tyrosine], red) and the changes in concentration of HPA (Δ[HPA], purple) and *p*‐cresol (Δ[*p*‐cresol], blue) in the tyrosine‐supplemented cultures.

To investigate its tyrosine metabolism, *C. scatologenes* was grown in modified LPBM medium [30] containing various amino acids required for growth of this bacterium, with proline as the Stickland acceptor, and supplemented with tyrosine as the Stickland donor. Supplementation with tyrosine led to an increase in final cell density (Figure 4a), and was accompanied by induction of *hpdJ, E, K* and *B*, as detected by qPCR (Figure 4b). The production of HPA (Figure 4c,d) and *p‐*cresol (Figure 4e,f) was observed in cultures containing tyrosine, but not in cultures lacking tyrosine, indicating that tyrosine is the precursor of both HPA and *p‐*cresol. Growth of the tyrosine‐containing culture was monitored over time, and found to correlate with consumption of tyrosine and corresponding production of HPA and *p‐*cresol in the media. HPA levels initially rose and later decreased, while *p*‐cresol production experienced a lag phase before rising (Figure 4g,h). This pattern suggests that tyrosine is initially converted into HPA, which is then transformed into *p*‐cresol during a later stage of the culture. A similar phenomenon was previously observed in experiments with *C. difficile* [31, 32].

### 2.7 Sequence Diversity of PFOR Homologs in the Order *Peptostreptococcales*

**FIGURE 5:**
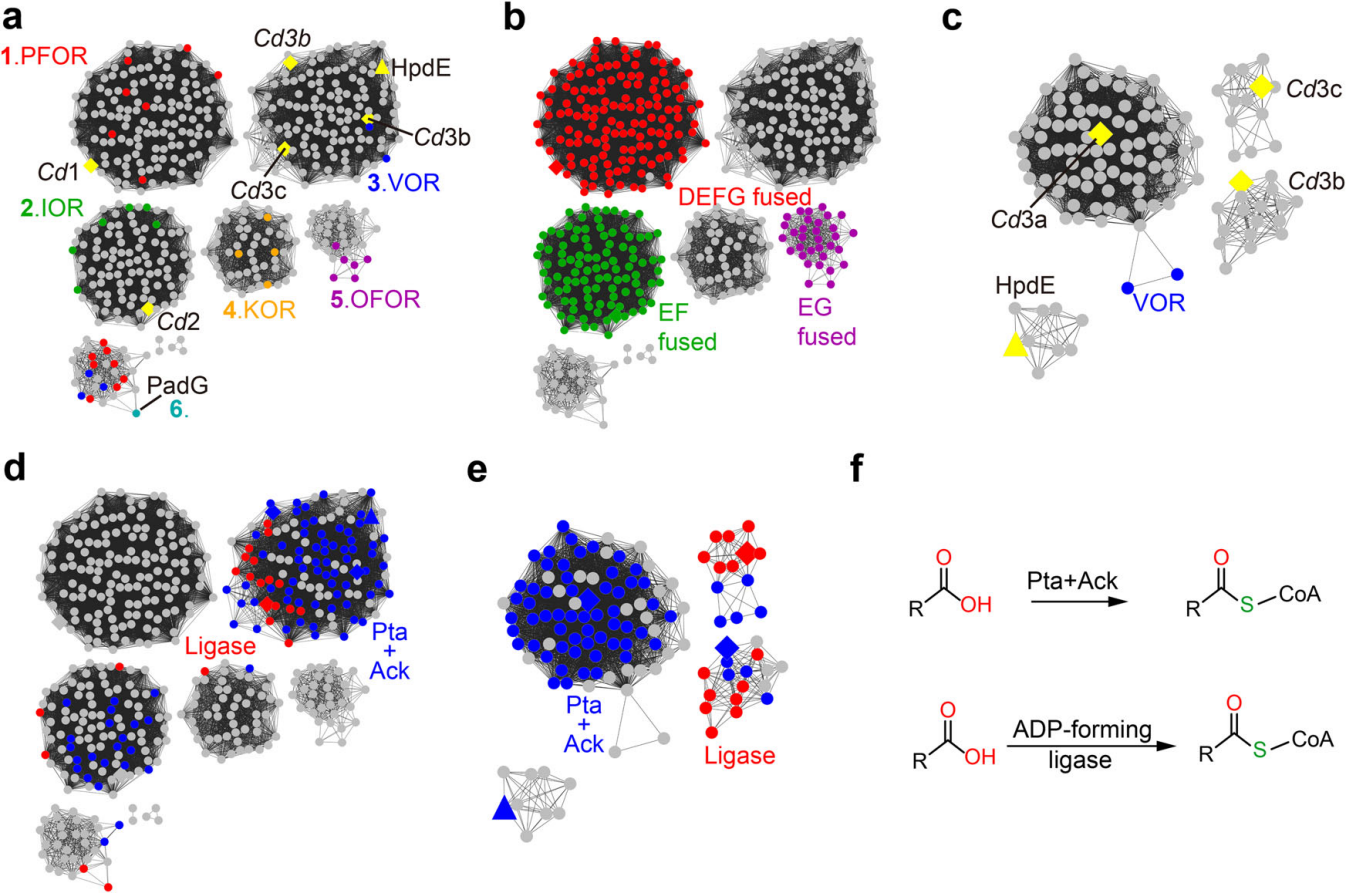
Bioinformatics analysis of bacterial PFOR homologs. a) Sequence Similarity Network (SSN) of PFOR homologs in the bacterial order Peptostreptococcales, along with several known PFOR homologs, at edge cutoff 10^−80^. Known PFOR homologs are colored according to their substrates: pyruvate (PFOR, red), linear and branched alkylpyruvates (VOR, blue), arylpyruvates (IOR, green), *α*‐ketoglutarate (KOR, orange), both pyruvate and *α*‐ketoglutarate (OFOR, purple), phenylglyoxalate (PadG, teal). Yellow diamonds denote enzymes from *Clostridium difficile* strain 630, and the yellow triangle denotes HpdE characterized in this paper. b) SSN colored according to subunit structure. c) SSN of cluster 3 from panel a) with edge cutoff increased to 10^−105^ d) SSN colored according to the presence of various enzymes in the 10‐open reading frame (10‐ORF) genome neighborhood: both phosphate acyltransferase and acyl kinase (Pta+Ack, blue), acyl‐CoA ligase (ligase, red). e) SSN of cluster 3 from panel c, with edge cutoff increased to 10^−105^. f) Scheme for possible alternative pathways for metabolism of the acyl‐CoA product.

Having characterized the enzymes in the tyrosine fermentation gene cluster of the environmental bacterium *C. scatologenes*, we then conducted a bioinformatics study to identify similar gene clusters in bacteria in the order *Peptostreptococcales*, which includes many amino acid‐fermenting bacteria such as *C. difficile, Clostridium sticklandii*, *Peptostreptococcus anaerobius* and *Emergencia timonensis*. A Sequence Similarity Network (SSN) was generated using Enzyme Function Initiative (EFI) webtools [33], containing 452 sequences belonging to the PFOR family (Pfam PF01855) within the *Peptostreptococcales*, along with *C. scatologenes* HpdE and other known PFOR family sequences from the SwissProt database. The network was visualized using CytoScape [34]. At an edge cutoff of 10^−80^, the sequences separate into six major clusters, each proposed to contain functionally related enzymes (Figure 5a).

Cluster 1 includes known pyruvate:Fdx oxidoreductases (PFORs) and is linked to an EKR domain (IPR019456), a hallmark of PFOR. Cluster 2 contains known indolepyruvate / phenylpyruvate / *p‐*hydroxyphenylpyruvate:Fdx oxidoreductases (IORs, family IPR017721). Cluster 3 contains known 2‐ketoisovalerate oxidoreductases (VORs) and *C. scatologenes* HpdE. Cluster 4 contains known 2‐ketoglutarate:Fdx oxidoredutases (KORs). Cluster 5 contains previously characterized broad specificity 2‐oxoacid:Fdx oxidoreductases (OFORs). Cluster 6 contains diverse enzymes, including PFOR, VOR and phenylglyoxalate dehydrogenase (PadG). The different clusters differ in their subunit composition. While clusters 3, 4 and 6 have the same subunit structure as HpdDEFG (D+E+F+G), subunits E and F are fused in cluster 2, subunits E and G are fused in cluster 5, and all four subunits are fused in cluster 1 (Figure 5b). Many of the cluster 2 and 3 sequences are located in proximity to either phosphate acyltransferase (PF01515) and acyl kinase (PF00871), or ADP‐forming acyl‐CoA ligase (PF13607), suggesting potential alternative pathways for metabolism of acyl‐CoA product (Figure 5d–f). Although PFOR homologs are challenging to reconstitute and assay, the kinases and ligases are simpler to study and can offer insights into the substrates of the associated enzymes.

### 2.8 Assays of Kinases and Ligases Associated with PFOR Homologs in *C. difficile*

**FIGURE 6:**
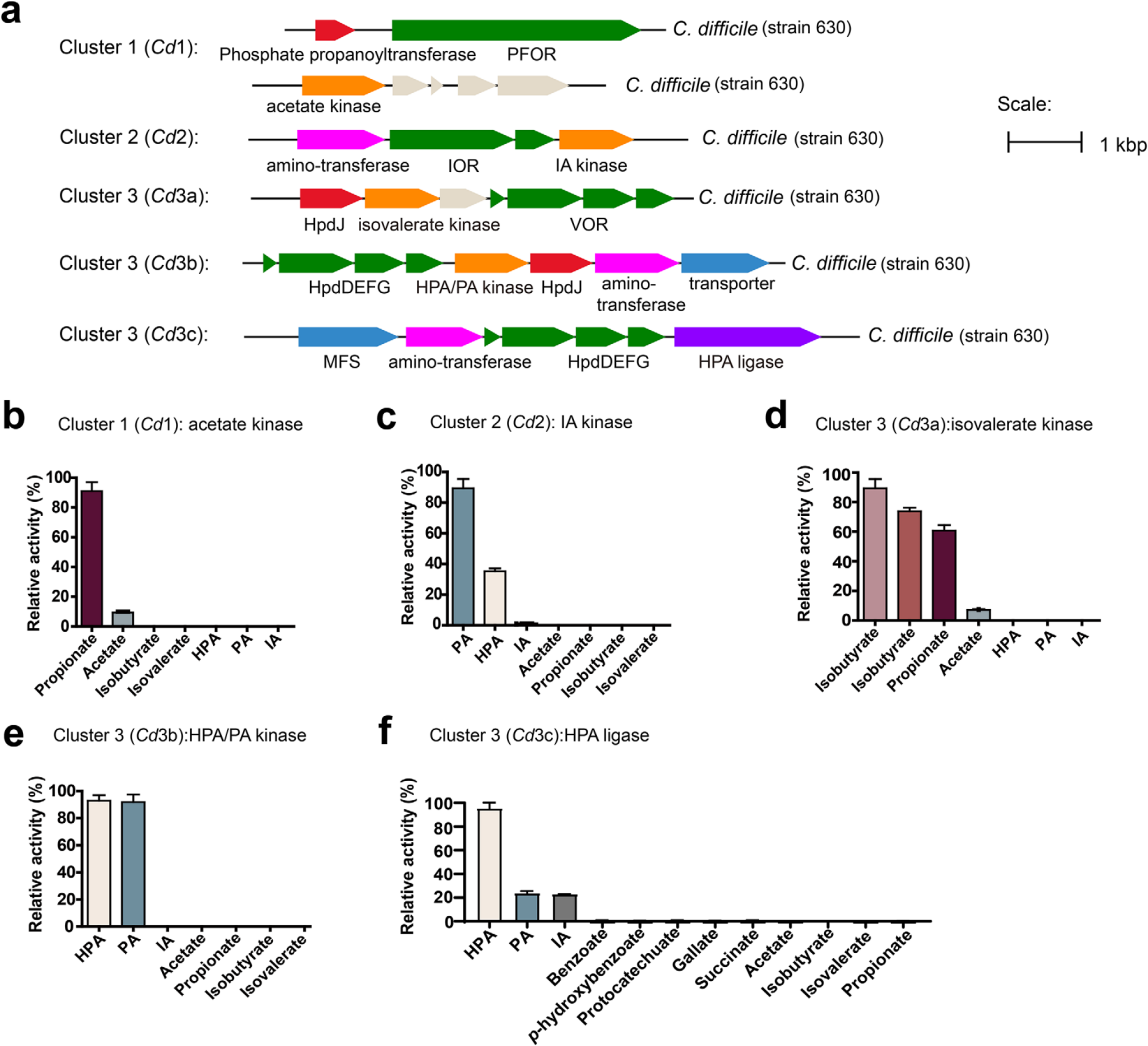
Gene clusters and relative catalytic activity (*k_cat_*) of kinases and ligase in *C. difficile*. a) Gene clusters for *C. difficile* containing the kinases and ligases assayed, including cluster 1, cluster 2, and cluster 3 (subdivided into 3a, 3b, and 3c). b–f) *k_cat_*‐based relative activity of acetate, IA, isovalerate, HPA/PA kinases and HPA ligase with different substrates.

We next focused on the key amino acid‐metabolizing pathobiont *C. difficile*. This bacterium contains five PFOR homologs, including a putative PFOR (*Cd*1, cluster 1), IOR (*Cd*2, cluster 2), and three more oxidoreductases of unknown specificity belonging to cluster 3 (*Cd*3a‐c). To further investigate the enzymes in cluster 3, the edge cutoff was increased to 10^−105^, causing cluster 3 to segregate into four subclusters. Three of the subclusters contain a sequence from *C. difficile* (*Cd*3a‐c), while the fourth contains *C. scatologenes* HpdE (Figure 5c). To explore potential substrate of the PFOR homologs, the kinases and ligases from *C. difficile* were assayed and their Michaelis‐Menten rate constants calculated (Figure 6; Figure S10, and Table S2). The known *C. difficile* acetate kinase (Ack) showed activity with both acetate and propionate (Figure S10a). *Cd*2 clusters with known IORs, and the kinase associated with *Cd*2 showed highest activity for PA and HPA, with lower but still significant activity for IA (Figure S10b), suggesting a link to arylpyruvate metabolism. *Cd*3a clusters with known VORs, and the kinase associated with *Cd*3a showed highest activity toward propionate, isobutyrate and isovalerate (Figure S10c), suggesting a link to (branched) alkylpyruvate metabolism. The kinase associated with *Cd*3b showed activity with PA and HPA, suggesting a link to (hydroxy)phenylpyruvate metabolism (Figure S10d). The ligase associated with *Cd*3c showed activity with PA, HPA and IA (Figure S10e), suggesting a link to arylpyruvate metabolism. In summary, the bioinformatics analyses and activity assays of associated kinases and ligases suggest that *C. difficile* contains PFOR, IOR, VOR, one potential (H)POR and one potential arylpyruvate oxidoreductase, although further experiments are needed to verify the substrates.

## 3 Discussion

Our experiments on enzymes encoded by the HPAD‐containing gene cluster in *C. scatologenes* demonstrate a pathway for conversion of tyrosine to HPA, involving HPA: ferredoxin oxidoreductase HpdDEFG, phosphate HPA‐transferase HpdJ and HPA kinase HpdK (Figure 1b). In vitro reconstitution of the anaerobic pathway demonstrated its ability to convert HPP to HPA, followed by decarboxylation to *p‐*cresol. Growth of *C. scatologenes* on tyrosine was accompanied by the accumulation of first HPA and then *p‐*cresol, along with induction of the genes under study, suggesting their relevance in vivo. This pathway for tyrosine fermentation is analogous to the oxidative branch of the Stickland pathway for alanine fermentation, involving the well‐studied enzymes PFOR, phosphate acetyltransferase Pta, and acetate kinase Ack, and is coupled to generation of ATP and reduced Fdx [17]. The use of reduced Fdx for energy production, through membrane‐based redox‐coupled ion translocation or cytosolic electron bifurcation mechanisms [35], is a feature of the energy metabolism in Clostridia. Conversion of aromatic amino acids to arylacetates is also known in Proteobacteria but uses a different mechanism for arylpyruvate metabolism involving TPP‐dependent arylpyruvate decarboxylases such as IP decarboxylase in *Enterobacter cloacae* [36], and PP decarboxylase in *Achromobacter* Eurydice [37] and *Proteus mirabilis* [38].

The recombinant production, purification, anaerobic reconstitution, and kinetic assays of the PFOR family enzyme HpdDEFG provided biochemical insights into arylpyruvate oxidation, a crucial step in aromatic amino acid fermentation in strict anaerobic bacteria. Although assays demonstrate its specificity for (hydroxy)phenylpyruvate, HpdDEFG is more closely related to previously characterized 2‐ketoisovalerate oxidoreductases (VORs), and distantly related to arylpyruvate oxidoreductases (IORs). VOR and IOR have been purified and biochemically studied from the fermenting archaeon *Pyrococcus furiosus*, where it is involved in amino acid fermentation, and from the methanogenic archaeon *Methanobacterium thermoautotrophicum*, where it is proposed to operate in reverse for amino acid biosynthesis [39, 40]. The IORs are active on phenyl‐, hydroxyphenyl‐ and indole‐pyruvate, while the *M. thermoautotrophicum* VOR is active on 2‐oxovalerate, with a lower activity on phenylpyruvate (45% compared to 2‐oxovalerate). In the polysaccharide‐fermenting gut bacterium *Bacteroides thetaiotaomicron*, IOR has recently been shown to be involved in conversion of phenylalanine to phenylacetate and its derivatives [41]. IOR in *Bacteroides* species colocalizes with an AMP‐forming ligase, and our assays confirm that it is an arylacetate‐CoA ligase (Figure S11). Since AMP‐forming ligases typically favor acyl‐CoA synthesis over degradation, the IOR in *Bacteroides* may play a role in aromatic amino acid biosynthesis, similar to methanogenic archaea, rather than in degradation.

The oxidative Stickland pathway for tyrosine metabolism in *C. scatologenes* contrasts with the reductive Stickland pathway for aromatic amino acid metabolism in the model amino acid‐fermenting bacterium *C. sporogenes*, which converts aromatic amino acids to phenyl‐, hydroxyphenyl‐, and indole‐propionate. *C. sporogenes* contains VOR but not IOR, and metabolizes branched chain amino acids via the oxidative Stickland pathway involving VOR. The kinase associated with the VOR in *C. sporogenes* showed highest activity towards propionate, isobutyrate and isovalerate (Figure S12), similar to the VOR in *C. difficile*. *C. sporogenes* also produces small amounts of PA and HPA, though at much lower levels than the aryl‐propionates. PA and HPA production was abolished in the VOR deletion mutant [2], suggesting that they may result from promiscuous activity of VOR on arylpyruvates, as observed in the archaeal VOR [40].

Growth experiments of *C. scatologenes* on tyrosine provided key insights into the link between tyrosine fermentation and cresol production. The lag between HPA accumulation in the media and its decarboxylation by HPAD to *p‐*cresol suggests that the two processes are not tightly coupled. In *C. difficile*, it was previously shown that *hpdB* expression is not concomitant with tyrosine metabolism, but is induced in a dose‐dependent manner by elevated levels of HPA [31, 42]. Unlike in *C. scatologenes*, most HPAD sequences are not located near tyrosine fermentation genes. Furthermore, HPAD (Figure S13) and IAD are present in bacteria that do not ferment aromatic amino acids. An example is *Olsenella scatoligenes*, which produces *p*‐cresol / skatole from HPA / indoleacetate but not from the corresponding amino acids [16]. We previously noted that IAD tends to occur in bacteria that also contain HPAD [16], suggesting a common metabolic function for arylacetate decarboxylases. While the physiological or metabolic role of GRE arylacetate decarboxylases remains unknown, it was proposed that the intracellular decarboxylation reaction may play a role in establishing a proton gradient for energy production [15].

In conclusion, characterization of the tyrosine fermentation enzymes provides biochemical insight into how aromatic amino acids are fermented via the oxidative Stickland pathway. This highlights a major route for production of aromatic metabolites including *p*‐cresol, with effects on gut microbial communities. Identification of the genes involved enables broader bioinformatic investigation of aromatic metabolite production, including the involvement of cross‐feeding between amino acid‐fermenting and arylacetate decarboxylating bacteria in the gut microbiome.

## 4 Experimental Section

Lysogeny broth (LB) medium was prepared with yeast extract and tryptone purchased from Oxoid, England. Acetonitrile and acetic acid used for liquid chromatography‐mass spectrometry (LC‐MS) were high‐purity solvents from Concord Technology and Merck respectively. Water used in this work was ultrapure deionized water from Millipore Direct‐Q. TALON Cobalt resin was from Clontech. Chemical reagents were purchased from Sigma–Aldrich, J&K, HEOWNS, and Solarbio.

All protein purification chromatographic experiments were performed on an “ÄKTA pure” or “ÄKTA prime plus” FPLC machine equipped with appropriate columns (GE Healthcare). Protein concentrations were calculated from the absorption at 280 nm measured using a Thermo Scientific Nanodrop One. Anaerobic experiments were conducted in a Lab2000 glovebox (Etelux) under an atmosphere consisting of N_2_ with less than 5 ppm O_2_.

### 4.1 Plasmid Construction

DNA fragments containing codon‐optimized ORFs were synthesized and inserted into either pET‐28a(+) or the modified pET28 vectors HT containing a His_6_‐tag and a Tobacco Etch Virus (TEV) protease cleavage site followed by the construct of interest, and HMT containing a His_6_‐tag, maltose binding protein (MBP) and a TEV site followed by the construct of interest by General Biosystems (Anhui, China). For co‐expression of HpdEFG, the three genes were inserted into a single HT vector at the *Ssp*I site. The construct contained, in tandem, His_6_‐tagged *hpdG*, ribosome binding site 1 (RBS1), *hpdE*, ribosome binding site 2 (RBS2), and *hpdF*. For co‐expression of HpdBC, the two genes were inserted into a single HT vector at the *Ssp*I site. The construct contained, in tandem, His_6_‐tagged *hpdB*, ribosome binding site 2 (RBS2), and *hpdC*. The sequences of RBS1 and RBS2 are GATGTCGACTAGGAGGAATATAAA and CTTAAAAAATAAGGAGGATTACACT respectively, both containing the underlined Shine‐Dalgarno sequence. *hpdF*, *hpdJ*, *hpdBC* and *paaK* (PA‐CoA‐ligase, PCL, Gene ID WP_006491104.1) [28], were each inserted into HT vector, while *hpdD*, *hpdA* were inserted into the HMT vector. *hpdK* and *IPP1* (inorganic pyrophosphatase) fragments were inserted into pET‐28a(+) at the *Nde*I/*Xho*I sites [43].

### 4.2 Recombinant Protein Production and Purification

HpdA (uniprot: Q84F14), HpdBC (HpdB uniprot: Q84F16, HpdC uniprot: Q84F15), HpdD (uniport: A0A0E3K4×1), HpdEFG (HpdE uniport: A0A0E3GSJ0, HpdF uniport: A0A0E3MAJ6, HpdG uniport: A0A0E3K460), HpdF (HpdF uniport: A0A0E3MAJ6), HpdJ (uniport: A0A0E3MBU2), HpdK (uniport: A0A0E3M8U5), PCL (uniport: B4E7B5) and IPP1 (uniprot: P00817) were heterologously expressed in *Escherichia coli* BL21 (DE3) cells harbouring the plasmids HMT‐HpdA, HT‐HpdBC, HMT‐HpdD, HT‐HpdEFG, HT‐HpdF, HT‐HpdJ, pET‐28a(+)‐His_6_‐HpdK, HT‐PCL, and pET‐28a(+)‐His_6_‐IPP1, respectively. Transformants were grown in LB supplemented with 50 µg/mL kanamycin (typically 1 L in a 2.6 L flask) at 37°C while being shaken at 220 rpm. When OD_600_ reached ∼0.8, the temperature was decreased to 16°C and isopropyl *β*‐D‐1‐thiogalactopyranoside (IPTG) was added to a final concentration of 0.3 mm to induce the production of the proteins [16]. IPP1 expression was very robust and could be purified to high homogeneity with ammonium sulfate precipitation followed by one step of DEAE anion exchange column [44]. All other proteins were purified by a TALON cobalt affinity chromatography. After elution with elution buffer [20 mm Tris‐HCl, pH 7.5, 200 mm KCl, 5 mm *β*‐mercaptoethanol (BME) and 150 mm imidazole], proteins were precipitated with 70% ammonium sulfate, re‐dissolved in storage buffer [20 mm Tris‐HCl, pH 7.5, 200 mm KCl, 5% glycerol, and 5 mm BME] and desalted using a G25 column pre‐equilibrated with the same buffer. Proteins eluted from G25 column were flash‐frozen with liquid N_2_ in aliquots, and stored at −80°C. The purified proteins were examined by SDS‐PAGE on a 12% gel. Protein concentrations were calculated using the following extinction coefficients at 280 nm: MBP‐HpdA (ε_280_ = 124 220 m ^−1^cm^−1^), HpdBC (ε_280_ = 153 450 m ^−1^cm^−1^), MBP‐HpdD (ε_280_ = 73 800 m ^−1^cm^−1^), HpdEFG (ε_280_ = 51 340 m ^−1^cm^−1^, based on an estimated molar ratio of HpdE:HpdF:HpdG = 10:1:10 from the SDS‐PAGE gel), HpdF (ε_280_ = 17 420 m ^−1^cm^−1^), HpdJ (ε_280_ = 8,940 m ^−1^cm^−1^), HpdK (ε_280_ = 29 340 m ^−1^cm^−1^), PCL (ε_280_ = 41 370 m ^−1^cm^−1^) and IPP1 (ε_280_ = 49 390 m ^−1^cm^−1^).

### 4.3 [Fe‐S] Cluster Reconstitution for MBP‐HpdD / MBP‐HpdA / HpdBC

MBP‐HpdD (∼40 µm) / MBP‐HpdA (∼70 µm) / HpdBC (∼35 µm) was degassed on a Schlenk line and brought into the glovebox. The reconstitution buffer contained 100 mm Tris‐HCl, pH 7.5, and 10 mm dithiotheritol (DTT). A solution of ferrous ammonium sulfate (8 eq. for MBP‐HpdD and HpdBC, 12 eq. for MBP‐HpdA) was added, followed by a solution of sodium sulfide (8 eq. for MBP‐HpdD and HpdBC, 12 eq. for MBP‐HpdA). The mixture was incubated overnight at 4°C in a cooling‐heating block (Dry Bath H2O3‐100C, Coyote Bioscience, Beijing, China). A solution of EDTA (8 eq. for MBP‐HpdD and HpdBC, 12 eq. for MBP‐HpdA), was then added, and excess iron and sulfide removed by repeated concentration with a centrifugal filter unit (1.5 mL Ym‐3 Amicon, Millipore), and dilution with buffer containing 20 mm HEPES, pH 7.5 and 0.1 m KCl.

### 4.4 [Fe‐S] Cluster Reconstitution for HpdEFG

Purification of HpdGEF resulted in substoichiometric HpdF. Therefore HT‐HpdF was expressed and purified separately. Purified HT‐HpdF (∼50 µm) and HT‐HpdGEF (∼120 µm) were degassed on a Schlenk line and brought into the glovebox. HT‐HpdF was then digested with TEV protease (Molar ratio TEV:HpdF = 1:10) to remove the His_6_ tag from HpdF for 1 h at 4°C maintained by a cooling‐heating block in the glove box. HpdF was then mixed with HT‐GEF to a final concentration of ∼30 µm with molar ratio of the three subunits estimated by SDS PAGE gel at roughly 1:1:1. A solution of ferrous ammonium sulfate was added, followed by a solution of sodium sulfide (4 eq.). A solution of EDTA (4 eq.) was then added, and excess of iron and sulfide removed by repeated concentration with a centrifugal filter unit (1.5 mL Ym‐10 Amicon, Millipore), and dilution with buffer containing 20 mm HEPES, pH 7.5 and 0.1 m KCl.

### 4.5 HpdDEFG Activity Assay

MBP was cleaved from reconstituted MBP‐HpdD by TEV protease digestion at 4°C for 1 h in the glove box just before the enzyme assays (Molar ratio TEV:HMT‐HpdD = 1:20). A 500 µL reaction mixture containing 50 mm Tris‐HCl pH 7.5, 5% glycerol, 2.5 mm MgCl_2_, 2 mm DTT, 2 mm CoASH, 2 mm benzyl viologen, 0.4 mm TPP, 10 mm HPP / PP / IPP/ pyruvate potassium salt, and 10 µm HpdDEFG (a mixture of HpdD and reconstituted HpdEFG in a 1:1 ratio), was incubated at room temperature (RT) for 4 h in the glovebox. Negative controls omitting either HPP or HpdDEFG were also performed. Reduced benzyl viologen exhibits a blue / purple color and was quantitated by absorbance at 555 nm [26].

### 4.6 Kinetic Assays

The spectrophotometric kinetic assays (assay volume 100 µL) for HpdDEFG activity were conducted at RT in a 1 cm Eppendorf cuvette using the cuvette mode of the Thermo Scientific Nanodrop OneC in the glovebox. The absorbance of each assay mixture was monitored at 555 nm, at 2 s intervals. To obtain the Michaelis‐Menten kinetic parameters, HPP, PP and IP assays were performed with varied substrate concentrations and a fixed enzyme concentration of 10, 20, 250 nm HpdDEFG respectively.

### 4.7 LC‐MS Detection of HPA‐CoA Formation

To produce HPA‐CoA as a molecular standard for LC‐MS analyses, a 400 µL reaction mixture containing 200 mm Tris‐HCl pH 7.5, 10 mm MgCl_2_, 40 mm HPA potassium salt, 2 mm ATP, 2 mm CoASH, and 0.1 mg/mL PCL, was incubated at 37°C for 30 min.

For detection of HPA‐CoA by LC‐MS, HpdDEFG reaction was stopped in a boiling water‐bath for 1 min. The precipitated protein was removed by centrifugation at 14 000 ×*g* for 1 min and the supernatant was filtered through a 0.22 µm PES (polyethersulfone) membrane. A 20 µL portion of the supernatant was analyzed by an Agilent 6420 Triple Quadrupole LC/MS instrument (Agilent Technologies) on a C18 column (Advantage ECHELON C18 4 µm 150 × 2.1 mm P/N: ADV8010, manufactured by ANALYTICAL). The solvent system consisted of 10 mm ammonium acetate and 0.001% acetic acid in H_2_O (A), and acetonitrile (B). The sample was eluted with a linear gradient of 1–20% B for 20 min with a flow rate of 0.5 mL/min. Elution was monitored by UV absorption at 254 nm. The HPA‐CoA product was verified by comparison with the standard produced by the PCL reaction, and by mass spectrometry.

### 4.8 Activity Assay for HpdJ

The substrates HPA‐CoA and PA‐CoA were produced enzymatically using PCL as described above in the presence of IPP1 to drive the reaction to completion. A 200 µL reaction mixture containing 100 mm Tris‐HCl pH 7.5, 15 mm KCl, 5 mm MgCl_2_, 10 mm HPA / PA, 3 mm ATP, 2 mm CoASH, 4 µm IPP1, and 5 µm PCL was incubated at RT for 30 min. The complete consumption of CoASH was confirmed by a DTNB assay. The 2 mm solution of HPA‐CoA / PA‐CoA was used without further purification for the subsequent HpdJ activity assays. In a 200 µL reaction mixture containing 0.1 m phosphate buffer pH 8.0, 0.1 mm HPA‐CoA / PA‐CoA, 0.1 mm DTNB, 1, 2, or 4 nm HpdJ was added to initiate the reaction. Absorbance at 412 nm was monitored at 6 s intervals to quantitate the sulfydryl contents upon release of CoASH. To obtain the Michaelis–Menten kinetic parameters, assays were performed with varying substrate concentrations and a fixed enzyme concentration of 2 nm HpdJ.

### 4.9 Activity Assay for HPA Phosphorylation by HpdK

ADP produced was detected using a pyruvate kinase and lactate dehydrogenase (PK‐LDH) assay kit purchased from Promega (Catalog # P0294). A typical enzyme assay contains 200 mm Tris‐HCl, pH 7.5, 10 mm MgSO_4_, 1 mm ATP, 0.4 mm NADH, 2 mm pyruvate enolphosphate, 10 units of PK‐LDH in a 200 µL of total volume at RT. Absorbance at 340 nm was monitored, and consumption of NADH was calculated based on the extinction coefficient 6300 m ^−1^ cm^−1^ [45]. For enzyme dose‐dependent assays, the enzyme concentrations were varied, in the presence of saturating concentrations (>5 times of K_M_) of the potassium salts of HPA, PA, or IA. To obtain the Michaelis–Menten kinetic parameters, substrate concentrations were varied, in the presence of a fixed enzyme concentration (0.005, 1, 10 µm of HpdK for HPA, PA and IA assays respectively, to account for the difference in *k*_cat_ with the different substrates).

### 4.10 LC‐MS Detection of HPA Formation

A 500 µL reaction mixture containing 10 µm HpdDEFG, 10 µm HpdJ, 10 µm HpdK, 50 mm Tris‐HCl, pH 7.5, 5% glycerol, 2 mm DTT, 2 mm CoASH, 2 mm ADP, 5 mm KH_2_PO_4_, 2 mm benzyl viologen, 0.4 mm TPP, 2.5 mm MgCl_2_ and 10 mm HPP potassium salt was incubated at RT for 4 h in the glovebox. Negative controls omitting HpdJ, HpdDEFG, all enzymes, or the substrate, HPP were also performed. The reaction was then stopped by incubation in a boiling water‐bath for 1 min. The precipitated protein was removed by centrifugation at 14 000 × *g* for 1 min and the supernatant was filtered through a 0.22 µm PES membrane. A 2 µL portion of the supernatant was analyzed by an Agilent 6420 Triple Quadrupole LC/MS instrument (Agilent Technologies) on a C18 column (Advantage ECHELON C18 4 µm 150 × 2.1 mm P/N: ADV8010, manufactured by ANALYTICAL). The solvent system consisted of 0.01% acetic acid in water (A) and acetonitrile (B). The samples were eluted with 5% B for 1 min, and 5%‐11.5% B gradient for 18 mins with a flow rate of 0.25 mL/min. The products were detected by extracted ion chromatographs, compared to standard sample, and further verified by mass spectra of eluted fraction of interest.

### 4.11 GC‐MS Detection of *p*‐cresol Formation

To produce *p*‐cresol as a molecular standard for GC‐MS analyses, a 300 µL reaction mixture containing 100 mm Tris‐HCl pH 7.5, 40 mm KCl, 5 mm MgCl_2_, 5 mm (NH_4_)_2_SO_4_, 5 mm cysteine, 0.2 mm Ti (III) citrate, 0.5 mm SAM, 10 µm HpdBC and 40 µm HpdA was incubated at 30°C for 15 min in the glovebox. Then 25 mm HPA was added. Full pathway reaction mixture containing 100 mm Tris‐HCl pH 7.5, 40 mm KCl, 2 mm CoA, 2 mm benzyl viologen, 2.5 mm MgCl_2_, 5 µm HpdDEFG, 0.4 mm TPP, 2.5 mm HPP, 2 mm ADP, 5 mm KH_2_PO_4_, 5 µm HpdJ, 5 µm HpdK, and activated 10 µm HPAD, was incubated at RT for 1 h in the glovebox. An equal volume of ethyl acetate was added and mixed with the samples. The organic phase was then filtered through a 0.22 µm filter and subjected to GC‐MS analysis, performed on a Shimadzu QP2010 GC‐MS system operating in ion scan mode (scan range: *m/z* 50–700), and equipped with a Rxi‐1 ms (30 m × 0.25 mm ID × 0.25 µm df) column. Helium was used as the carrier gas with a flow rate of 1.48 mL/min. The injector was operated in “splitless” mode with the injector temperature maintained at 250°C. The oven temperature for the Rxi‐1 ms column was ramped from 80 to 250°C at 15°C per min, and held 2 min. In total ion count (TIC) mode, the peak was observed at the retention time 9.82 min, corresponding to *p*‐cresol standard.

### 4.12 Growth of *C. scatologenes* and Real‐Time Fluorescence Quantitative PCR Analyses

*Clostridium scatologenes* (DSM 757) was purchased from the German Collection of Microorganisms and Cell Cultures GmbH (DSMZ, Leibniz Institute, Germany). Cells were first inoculated into DSM 110 medium and cultivated anaerobically at 37°C for 3 days. Then, 100 µL portions of the starter culture were transferred into two anaerobic bottles containing 5 mL modified LPBM medium [29] and each of the following _L_‐amino acids with a final concentration of 1 mm: glycine, valine, leucine, isoleucine, methionine, histidine, arginine and threonine. 2.5 mm tyrosine was added as energy resource for cell culture, and a negative control with no tyrosine added was also performed. To detect the product formation of *p*‐cresol, 500 µL of cultures were centrifuged at 10 000 × *g* for 10 min. The supernatant was extracted with equal volume of ethyl acetate. The upper organic phase was then subjected to GC‐MS analysis after filtered through a 0.22 µm filter. To detect the product formation of HPA, the supernatant was analyzed by LC‐MS on a ZIC‐HILIC column (5 mm, 200 Å, 150 × 4.6 mm; Merck) after filtered through a 0.22 µm filter. The LC conditions were as follows: 90% B to 65% B in 40 min, 65% B to 50% B in 5 min, to 90% B in 5 min. RNAs were extracted from ∼10^10^ cells from each culture using TRIzol reagent (Thermo Fisher). 1 µg RNA was revere transcribed using GoScript Reverse Transcription System (Promega) in 20 µL reaction mixture containing 0.5 µg random primers. For long‐term storage, obtained cDNA can be frozen at −80°C. Reversed transcriptase was inactivated at 70°C for 15 min prior to real‐time qPCR. In a typical qPCR reaction, 20 µL of reaction mixture contained 4 µL of 6 × diluted cDNA, 10 µm gene‐specific forward and reverse primers and 1 × SYBR Green GoTaq qPCR Master Mix (Promega). qPCRs were performed on a LongGene Q2000A (Hangzhou LongGene Scientific Instruments Co., Ltd., China). The primers were designed using primer3plus (http://www.primer3plus.com) (Table S1). All reagents and consumables for the experiments were RNase‐free. The data was analyzed by means of the ΔΔCt method and the values were normalized using 16S rRNA as an internal control.

### 4.13 Kinetic Assays for Kinases and Ligase Using Different Substrates

A typical enzyme assay contains 200 mm Tris‐HCl, pH 7.5, 10 mm MgSO_4_, 1 mm ATP, 0.4 mm NADH, 2 mm phosphoenolpyruvate, 10 units of PK‐LDH and 50 mm of the carboxylate substrate (HPA / PA / IA / acetate / isovalerate / isobutyrate / propionate) in a 200 µL of total volume at RT. Absorbance at 340 nm was monitored, and consumption of NADH was calculated based on the extinction coefficient 6300 m ^−1^ cm^−1^ [45]. To obtain the Michaelis–Menten kinetic parameters, substrate concentrations were varied, in the presence of a fixed enzyme concentration (0.3, 0.3, 1 µm of HPA/PA kinase for HPA, PA and IA assays respectively; 0.02, 0.01, 1 µm of IA kinase for HPA, PA and IA assays respectively; 20, 10 nm of acetate kinase for acetate / propionate assays respectively; 16, 16, 16, 200 nm of isovalerate kinase for isovalerate / isobutyrate / propionate / acetate assays respectively; 5, 5, 10 nm of isovalerate kinase (*C. sporogenes*) for isobutyrate / isovalerate / propionate assays respectively; 0.05, 0.2, 0.1 µm of HPA ligase (*C. difficile*) for HPA, PA and IA assays respectively), to account for the difference in *k*_cat_ with the different substrates).

### 4.14 LC‐HRMS for AMP‐Forming IA Ligases Using Different Substrates

To investigate the substrate specificity of IA ligase, enzymatic reactions were conducted using HPA, PA, and IA as substrates. A 200 µL reaction mixture containing 50 mm Tris‐HCl, pH 7.5, 10 µm IA ligase, 2 mm ATP, 2 mm CoA, 20 mm HPA / PA / IA, 10 mm MgCl_2_, 100 mm KCl was incubated at RT for 30 min. Negative controls were prepared by omitting ATP, CoA, HPA / PA / IA, or IA ligase. After incubation, proteins were removed by extraction with an equal volume phenol / chloroform / isopentanol (25:24:1). The aqueous phase was then collected via centrifugation, filtered through a 0.22 µm nylon membrane. The samples (10 µL) were subjected to liquid chromatography–high‐resolution mass spectroscopy (LC‐HRMS) analyses using a Q Exactive HF/UltiMate 3000 RSLCnano (Thermo Fisher) instrument equipped with an Xtimate C18 column (4.6 × 150 mm), using the same elution protocol for the LC‐MS assay for HpdDEFG as described above. The detailed ion source parameters were as follows: mass resolution, 120 000; spray voltage, 3.0 kV for negative; capillary temperature, 320°C; sheath gas flow rate (arb), 50; aux gas flow rate (arb), 15; probe heater temperature, 360°C; mass range (m/z), 100–1200.

## Author Contributions

L.J., Y.W., X.L., D.L., Z.L., Y.T., J.Y., A.N., M.X., M.H. and C.Z. conducted the experiments; L.J., Y.W., S.Y., Y.L., E.L.A., H.Z., and Y.Z. designed the experiments and wrote the paper.

## Funding

This work was supported by the National Natural Science Foundation of China (NSFC) Distinguished Young Scholar of China Program 32125002 (Y.Z.), the New Cornerstone Science Foundation NCI202321 (Y.Z.), National Natural Science Foundation of China (grant 31870049 and 31570060) (Y.Z.), and the Agency for Science, Research and Technology of Singapore Visiting Investigator Program (H.Z.).

## Conflicts of Interest

The authors declare no conflict of interest.

## Supporting information




**Supporting File**: advs75061‐sup‐0001‐SuppMat.docx.

## Data Availability

The data that support the findings of this study are available in the supplementary material of this article.
