# Supplementary material for: Enzymatic Basis for the Oxidative Branch of Aromatic Amino Acid Fermentation Leading to p‐cresol Formation
Source: Adv Sci (Weinh). 2026 Mar 31;13(34):e75061. doi: 10.1002/advs.75061 (PMC13285125; doi:10.1002/advs.75061)
Supplement: Supplementary file 1 — Supporting File: advs75061‐sup‐0001‐SuppMat.docx. [file ADVS-13-e75061-s001.docx]

**Supporting Information**

**Enzymatic basis for the oxidative branch of aromatic amino acid fermentation leading to *p*-cresol formation**

**Authors:** Li Jiang^1,2,3†^, Yifeng Wei^4†^, Xumei Liu^1^, Dazhi Liu^1^, Zhenyu Liu^5^, Yang Tong^1^, Jinyu Yin^1^, Ankanahalli N Nanjaraj Urs^1^, Meining Xing^1^, Mark A. Harrison^6^, Chuyuan Zhang^1^, Sheng Yang^7^, Yunzi Luo^5^, Ee Lui Ang^4^, Huimin Zhao^8*^, Yan Zhang^1,2,3,5,9*^

^1^New Cornerstone Science Laboratory, School of Pharmaceutical Science and Technology, Tianjin University, Tianjin 300072, China.

^2^Tianjin Key Laboratory for Modern Drug Delivery & High-Efficiency, Collaborative Innovation Center of Chemical Science and Engineering, School of Pharmaceutical Science and Technology, Tianjin University, Tianjin 300072, China.

^3^Frontiers Science Center for Synthetic Biology (Ministry of Education), Tianjin University, Tianjin 300072, China.

^4^Singapore Institute of Food and Biotechnology Innovation, Agency for Science, Technology and Research (A∗STAR), Singapore 138669, Singapore.

^5^Key Laboratory of Systems Bioengineering (Ministry of Education), School of Chemical Engineering and Technology, Tianjin University, Tianjin 300072, China.

^6^Department of Infection Biology, London School of Hygiene and Tropical Medicine, London, United Kingdom.

^7^Key Laboratory of Synthetic Biology, Center for Excellence of Molecular Plant Science, Chinese Academy of Sciences, Shanghai, China

^8^Department of Chemical and Biomolecular Engineering, University of Illinois at Urbana-Champaign, 600 South Mathews Avenue, Urbana, Illinois, 61801, USA.

^9^School of Life Sciences and Biotechnology, Shanghai Jiao Tong University, Shanghai, 200240, China.

^†^ These authors contribute to this work equally

^*^ *To whom correspondence should be addressed:*

Prof. Yan Zhang, New Cornerstone Science Laboratory, School of Pharmaceutical Science and Technology, Tianjin University, Tianjin 300072, China. Phone: (86) 22-87401835. Fax: (86) 22-87401830. E-mail: [yan.zhang@tju.edu.cn](mailto:yan.zhang@tju.edu.cn);

Prof. Huimin Zhao, Department of Chemical and Biomolecular Engineering, University of Illinois at Urbana-Champaign, 600 South Mathews Avenue, Urbana, Illinois, 61801, USA. Phone: (217) 333-2631. Fax: (217) 333-5052. E-mail: [zhao5@illinois.edu](mailto:zhao5@illinois.edu).

**Table S1. Primers used for qPCR experiments**

| **Primer name** | **Primer sequence 5'-3'** |
| --- | --- |
| HpdJ-F | GCAGCAGTAGTAGCAGCTCA |
| HpdJ-R | AGTTCTACTGCCTTTGCTGCT |
| HpdE-F | TTGCCTGAAGTTGGAGGGAA |
| HpdE-R | GGGCCTCCACGTGATATGTT |
| HpdK-F | GCTGCAGTTGTTGGAAGAGG |
| HpdK-R | ACCTTCATTGCCATTGCCCT |
| HpdB-F | GTTGGTGCTGGTGGAGGTAA |
| HpdB-R | ATTACTTCACGCCCCTGTGG |
| 16S rRNA-F | CACATGCAAGTCGAGCGATG |
| 16S rRNA-R | TGAGCCGTTACCTCACCAAC |

**Table S2. Kinetic parameters of kinases and ligase in *C. difficile***

| **Enzymes** | **Reaction substrates** | ***k_cat_* （s^-1^）** | ***K*_M_ (mM)** | ***k_cat_* / *K*_M_ (M·s^-1^)** |
| --- | --- | --- | --- | --- |
| *Cd*1: acetate kinase | Propionate | 402.1 ± 41.5 | 19.6 ± 4.2 | 2.1×10^4^ ± 5.0×10^3^ |
|  | Acetate | 30.7 ± 1.6 | 19.1 ± 3.4 | 1.6×10^3^ ± 3.0×10^2^ |
| *Cd*2: IA kinase | IA | 0.8 ± 0.02 | 1.2 ± 0.2 | 6.7×10^2^ ± 1.1×10^2^ |
|  | PA | 61.4 ± 3.5 | 5.5 ± 1.2 | 1.1×10^4^ ± 2.5×10^3^ |
|  | HPA | 22.1 ± 0.8 | 1.0 ± 0.2 | 2.2×10^4^ ± 4.5×10^3^ |
| *Cd*3a: Isovalerate kinase | Isovalerate | 104.3 ± 6.2 | 8.7 ± 1.7 | 1.2×10^4^ ± 2.5×10^3^ |
|  | Isobutyrate | 86.0 ± 2.4 | 3.8 ± 0.5 | 2.3×10^4^ ± 3.1×10^3^ |
|  | Propionate | 70.8 ± 3.7 | 14.8 ± 2.3 | 4.8×10^4^ ± 7.9×10^3^ |
|  | Acetate | 8.3 ± 0.4 | 52.9 ± 9.1 | 1.6×10^2^ ± 2.9×10^1^ |
| *Cd*3b: HPA / PA kinase | HPA | 2.2 ± 0.1 | 8.4 ± 1.1 | 2.6×10^2^ ± 3.6×10^1^ |
|  | PA | 2.2 ± 0.1 | 8.8 ± 1.6 | 2.5×10^2^ ± 5.8×10^1^ |
| *Cd*3c: HPA ligase | HPA | 16.7 ± 2.3 | 16.9 ± 6.7 | 9.9×10^2^ ± 4.2×10^2^ |
|  | PA | 5.9 ± 0.9 | 132.1 ± 41 | 4.5×10^1^ ± 1.6×10^1^ |
|  | IA | 4.6 ± 1.4 | 31.4 ± 21.8 | 1.5×10^2^ ± 1.5×10^2^ |


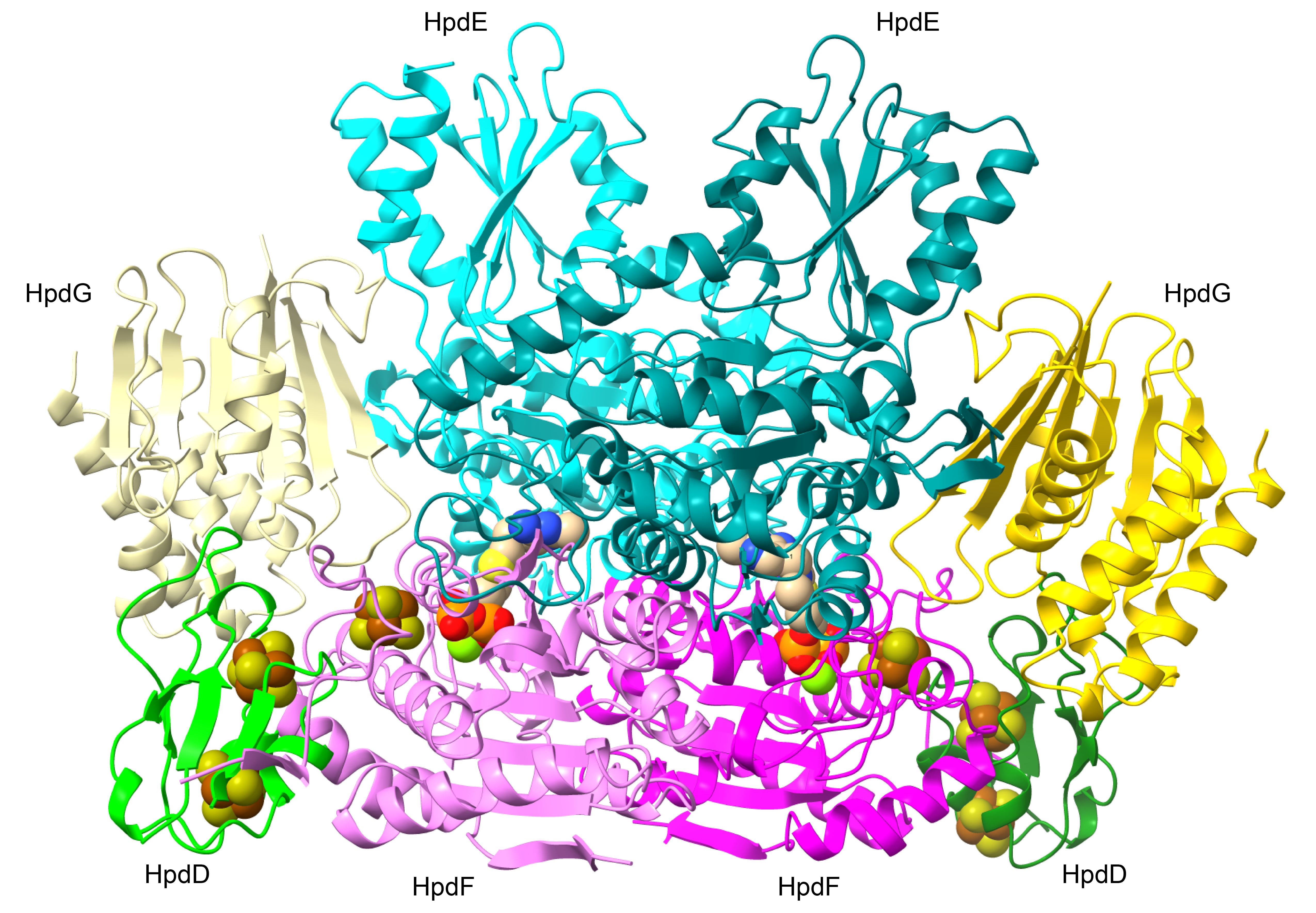


**Figure S1. AlphaFold3 structural model of HPP:Fdx oxidoreductase HpdDEFG.** The ligand positions were estimated by superimposing the model with *Desulfovibrio africanus* PFOR (PDB: 1B0P)[^23^](#_ENREF_37). While *Da*PFOR is a C2 symmetric homodimer, each protomer in the *C. scatologenes* enzyme is split into four ORFs, HpdDEFG. The [4Fe-4S] clusters (two coordinated by HpdD, one by HpdF) and the Mg^2+^-TPP cofactor are rendered in spheres. The four different subunits are displayed in different colours with HpdD in lime and green, HpdE in cyan and dark cyan, HpdF in violet and magenta, and HpdG in pale goldenrod and gold.


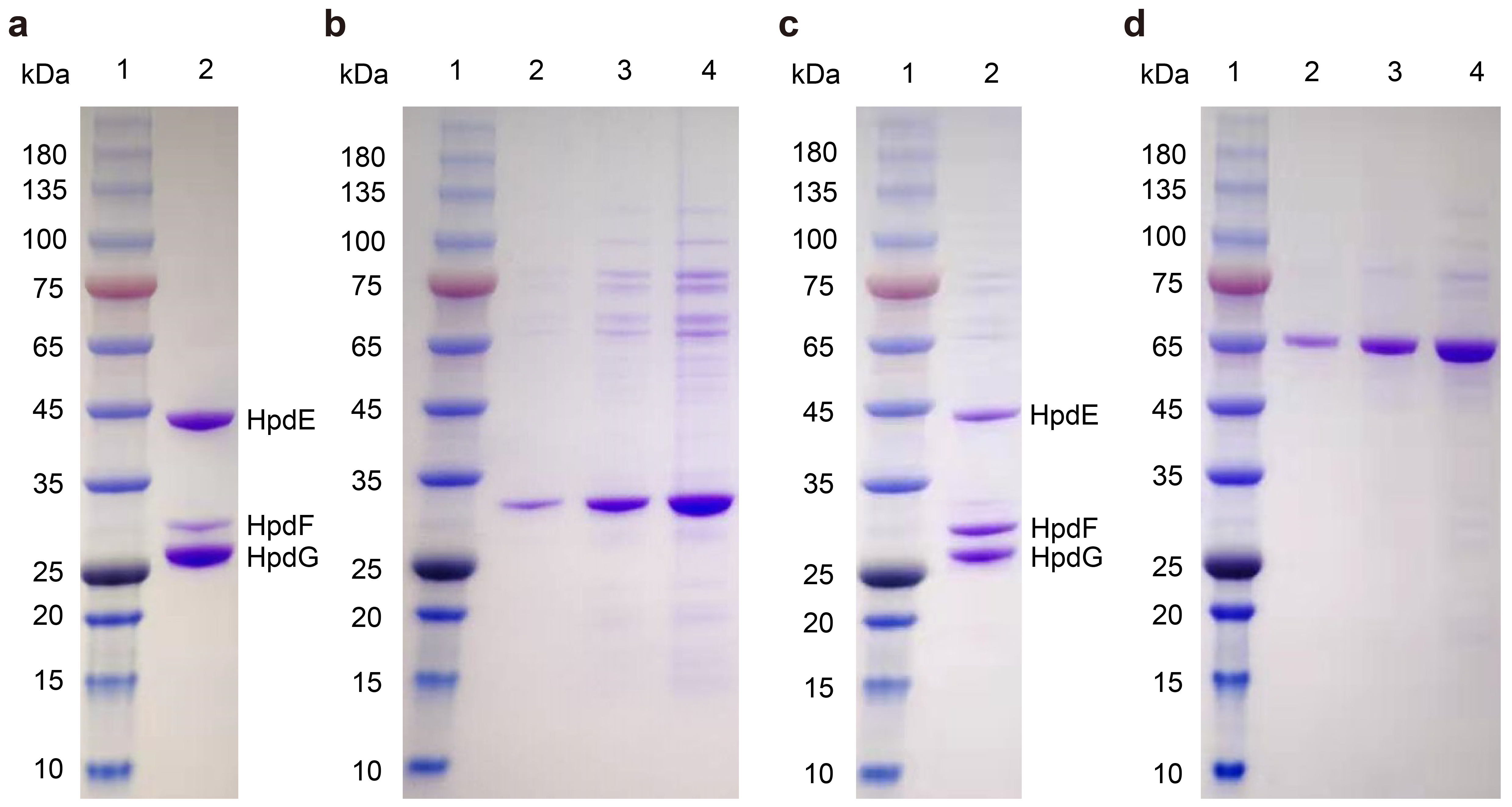


**Figure S2. SDS-PAGE of purified PFOR subunits.** **a.** Purified HT-HpdEFG with: lane1, molecular weight marker; lane 2, 4 µg of HT-HpdEFG. **b.** Purified HT-HpdF with: lane1, molecular weight marker; lane 2-4, 1, 2, 4 µg of HT-HpdF. **c.** Mixture of HpdEFG with equal molar subunit E, F, G. lane1, molecular weight marker; lane 2, 4 µg of EFG mixture. **d.** Purified HMT-HpdD with: lane1, molecular weight marker; lane 2-4, 1, 2, 4 µg of HMT-HpdD.


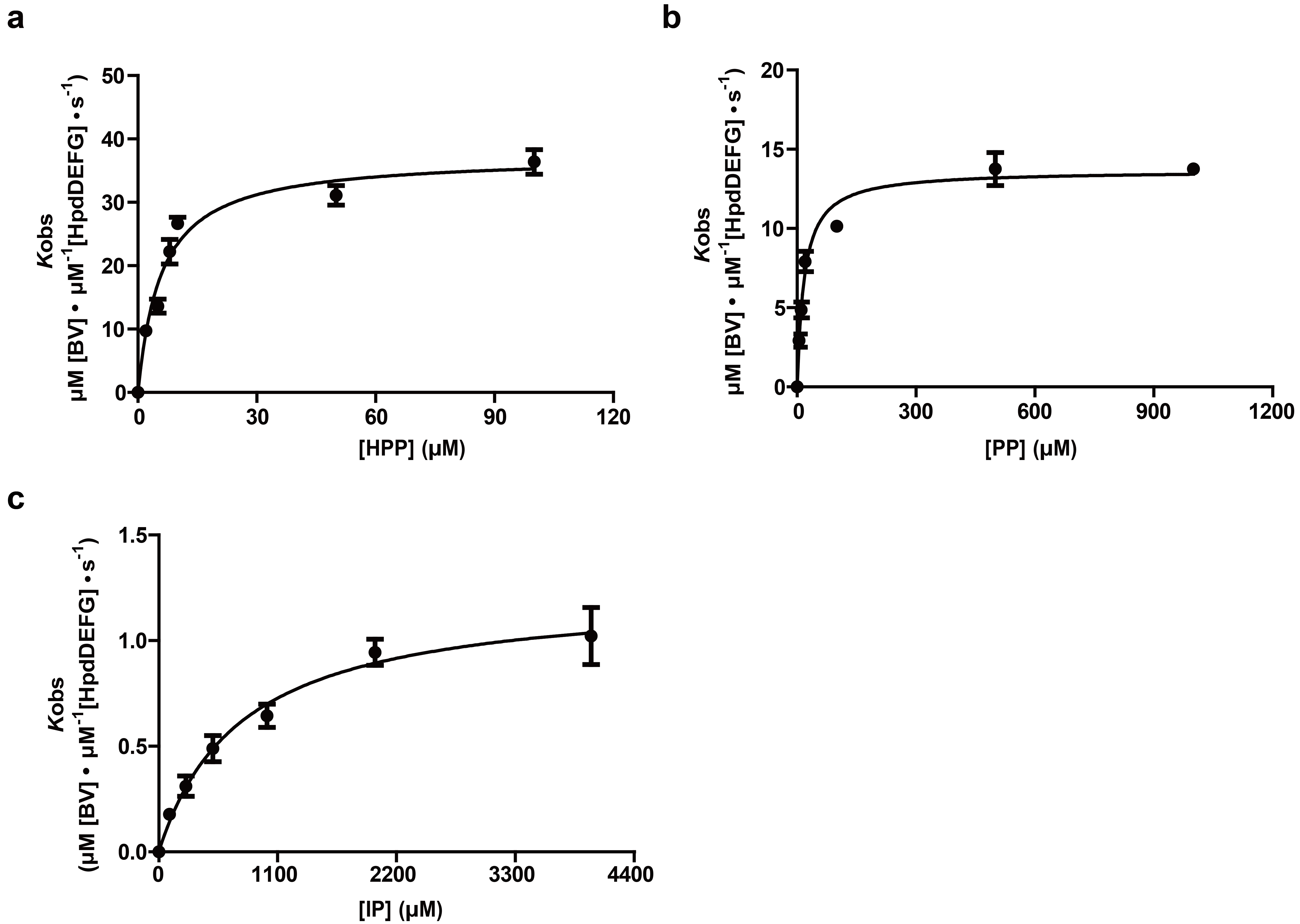


**Figure S3. Kinetic assays on HpdDEFG using different substrates.** Benzyl viologen (BV^2+^) was used as the electron acceptor following a previously reported procedure for 2-oxoglutarate:Fdx oxidoreductase[^26^](#_ENREF_19), which allows a colorimetric readout of enzyme activity through the formation of the blue-purple BV^+^. The rate of reaction was monitored by the increase of absorbance at 555 nm. **a.** HPA. **b.** PA and **c.** IA were used as substrate in the presence of 10 nM, 20 nM, and 250 nM HpdDEFG respectively.


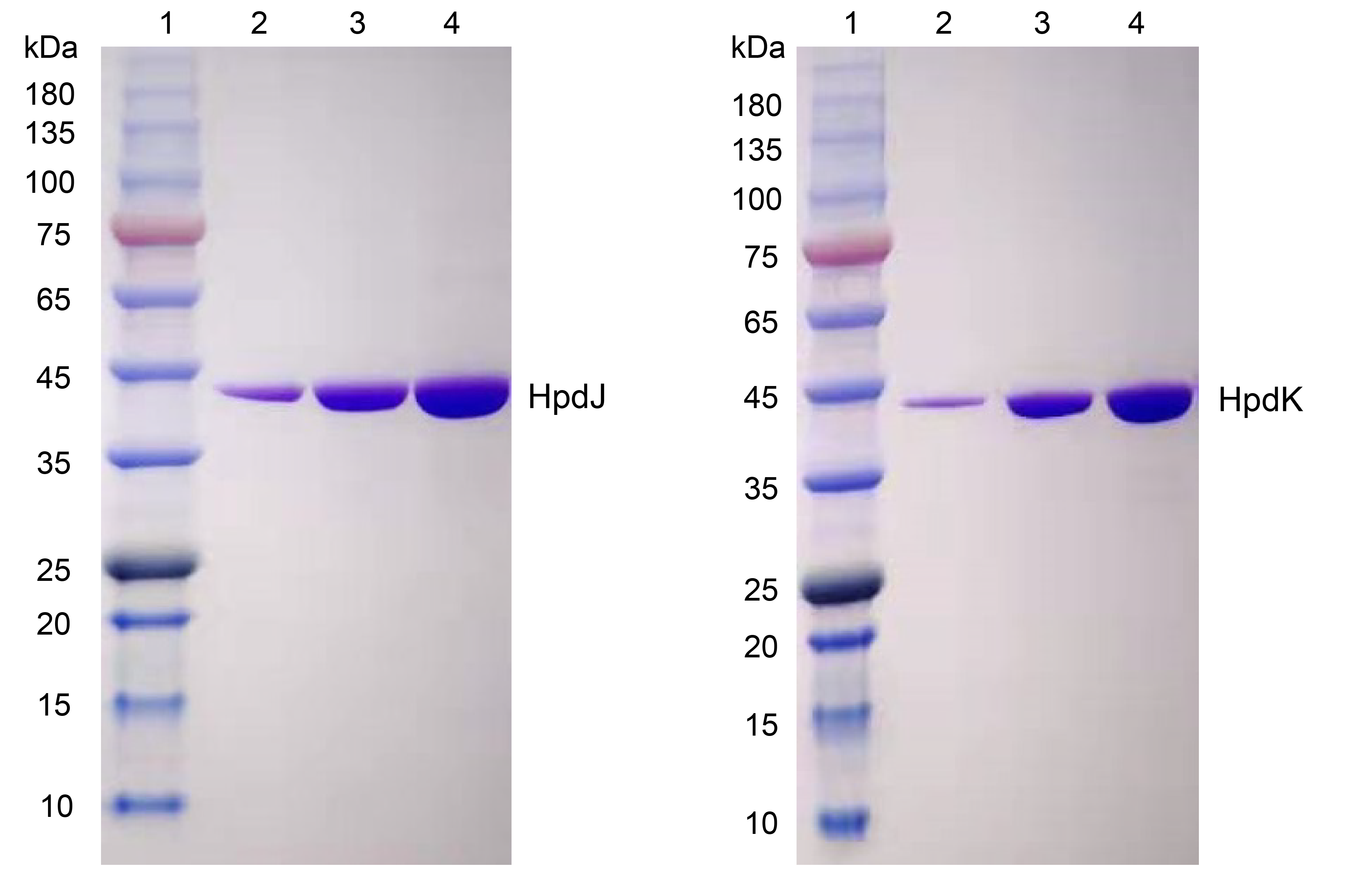


**Figure S4. SDS-PAGE of purified HpdJ and HpdK.** Purified HT-HpdJ with: lane1, molecular weight marker; lane 2-4, 1, 2, 4 µg of HT-HpdJ. Purified His_6_-HpdK with: lane1, molecular weight marker; lane 2-4, 1, 2, 4 µg of His_6_-HpdK. Both gels are 12% SDS gel.


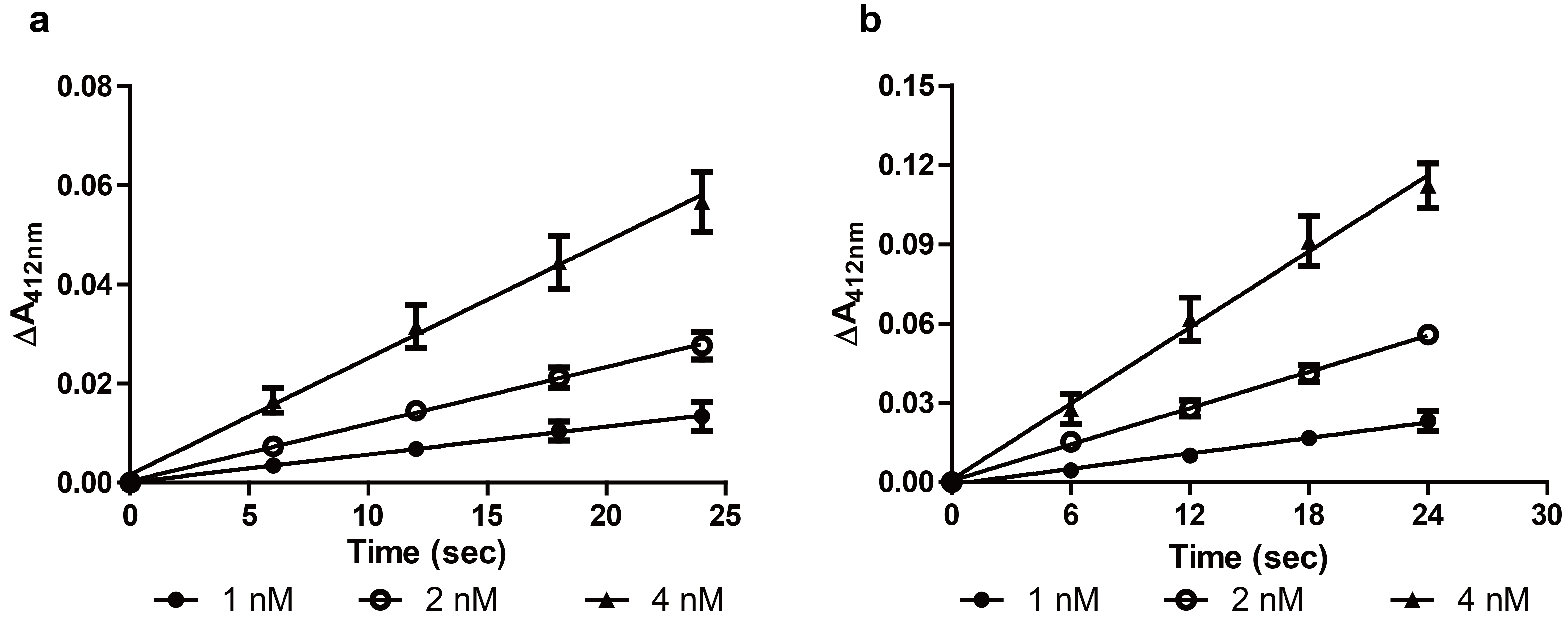


**Figure S5. Time- and enzyme dose-dependent spectroscopic assays of HpdJ activity. a.** HPA-CoA and **b.** PA-CoA were used as substrate. PCL was used to enzymatically generate HPA-CoA and PA-CoA for **a** and **b**. Assays monitoring the absorbance at 412 nm correlate the formation of sulfydryl group of CoASH.


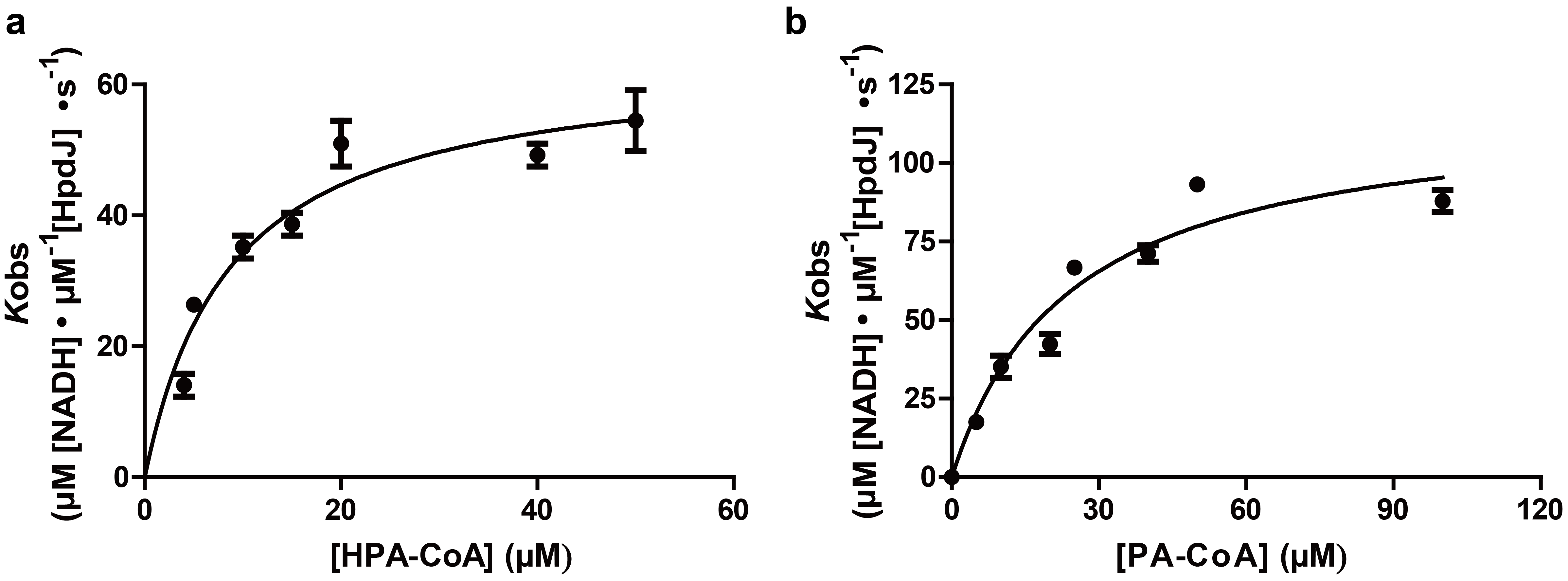


**Figure S6. Kinetic assays on HpdJ using different substrates.** The rate of reaction was monitored by measuring the increase of absorbance at 412 nm as CoASH is formed. **a.** HPA-CoA. **b.** PA-CoA generated enzymatically were used as substrate in the presence of 2 nM HpdJ.


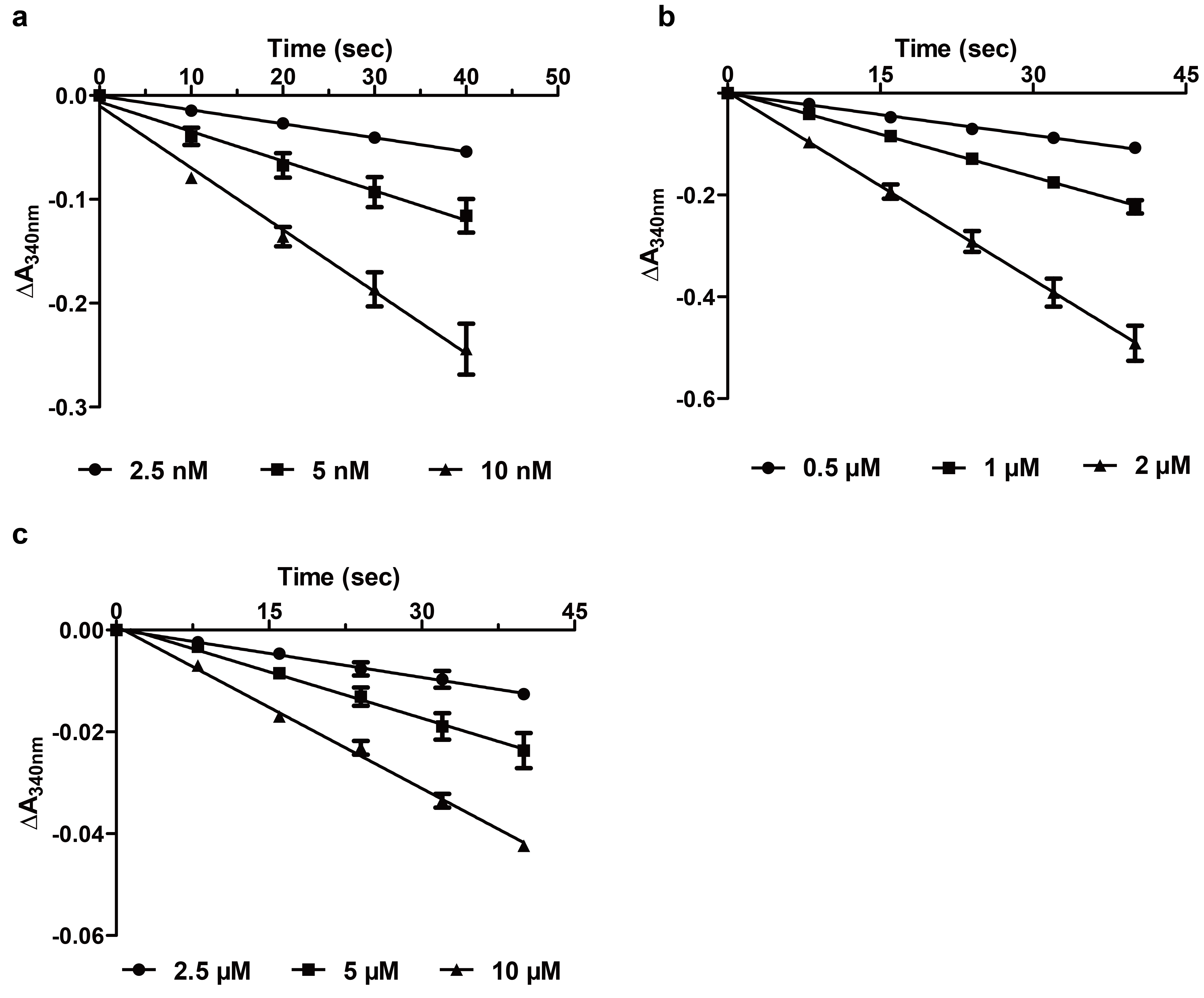


**Figure S7. Time- and enzyme dose-dependent spectroscopic assays of HpdK activity.** ADP formation was coupled by PK and LDH to the decrease of NADH. **a.** HPA. **b.** PA and **c.** IA were used as substrate. Assays monitoring the absorbance at 340 nm correlate the decrease of NADH.


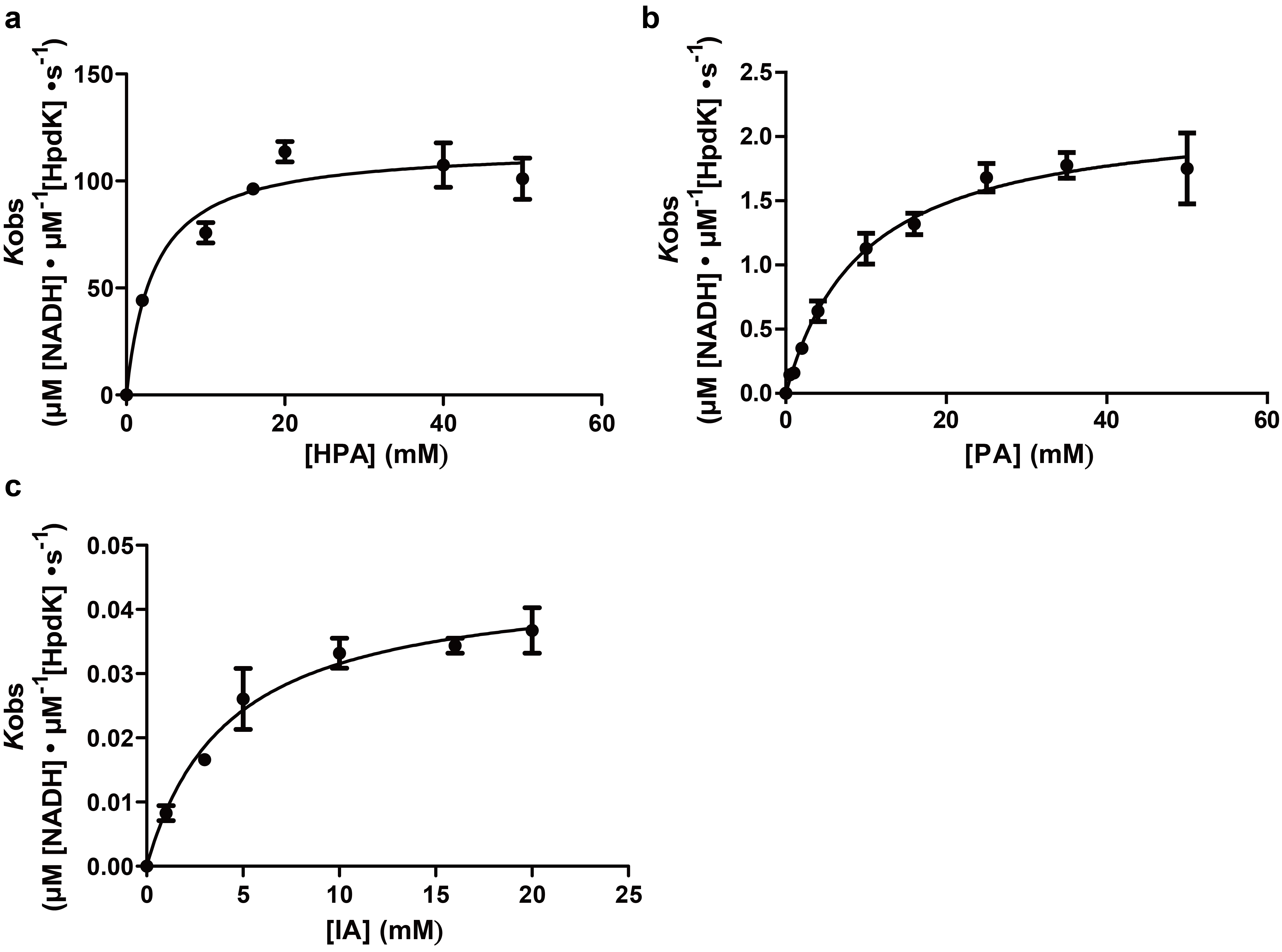


**Figure S8. Kinetic assays on HpdK using different substrates.** ADP formation was coupled by PK and LDH to the decrease of NADH. The rate of reaction was monitored by the decrease of absorbance at 340 nm. **a.** HPA. **b.** PA and **c.** IA were used as substrate in the presence of 5 nM, 1μM, and 10 μM HpdK respectively.


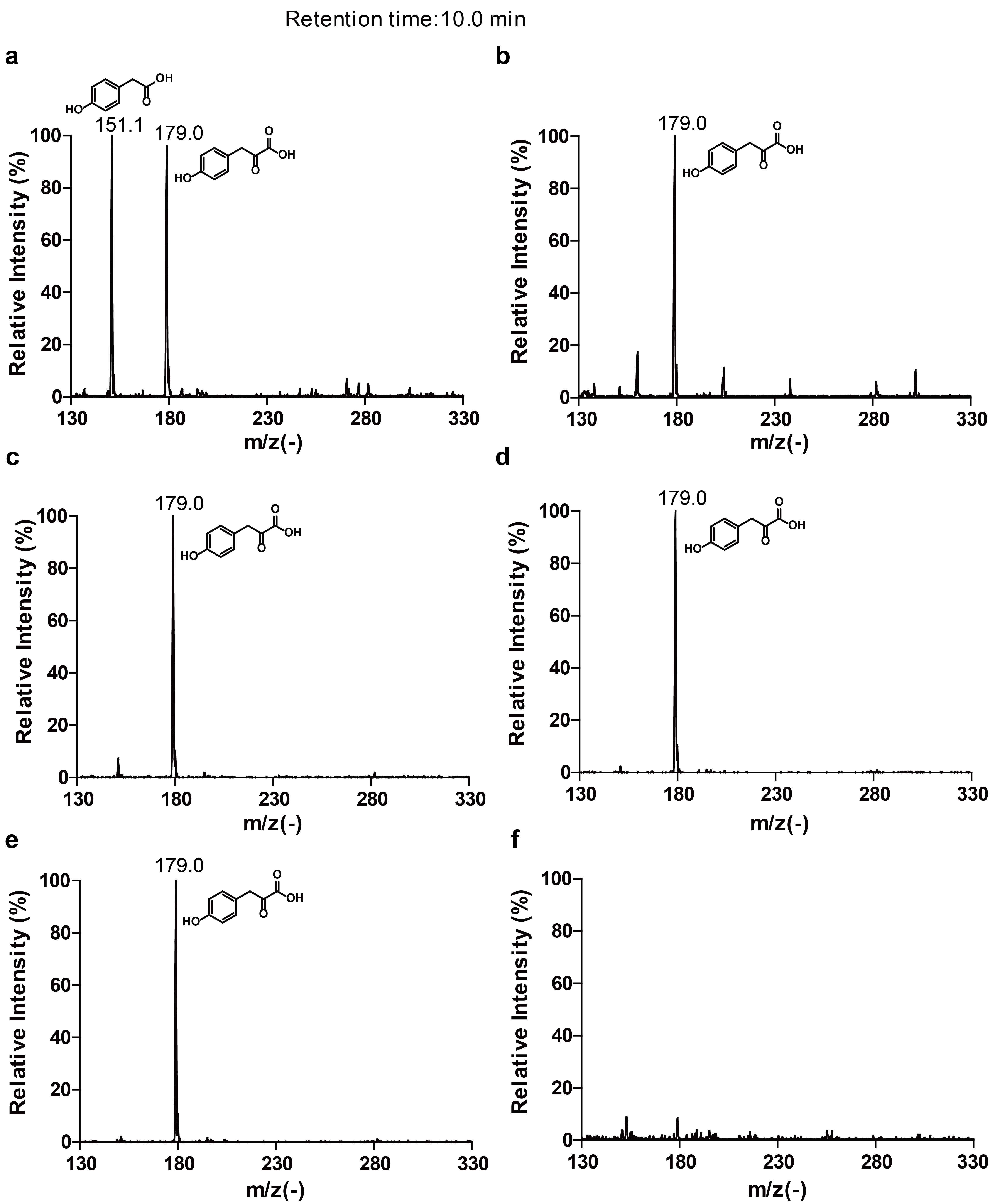


**Figure S9. Negative ionization mass spectra of the reconstituted pathway assay. a.** Complete assay. **b.** W/O HpdK. **c.** W/O HpdJ. **d.** W/O HpdDEFG. **e.** W/O enzymes. **f.** W/O HPP.


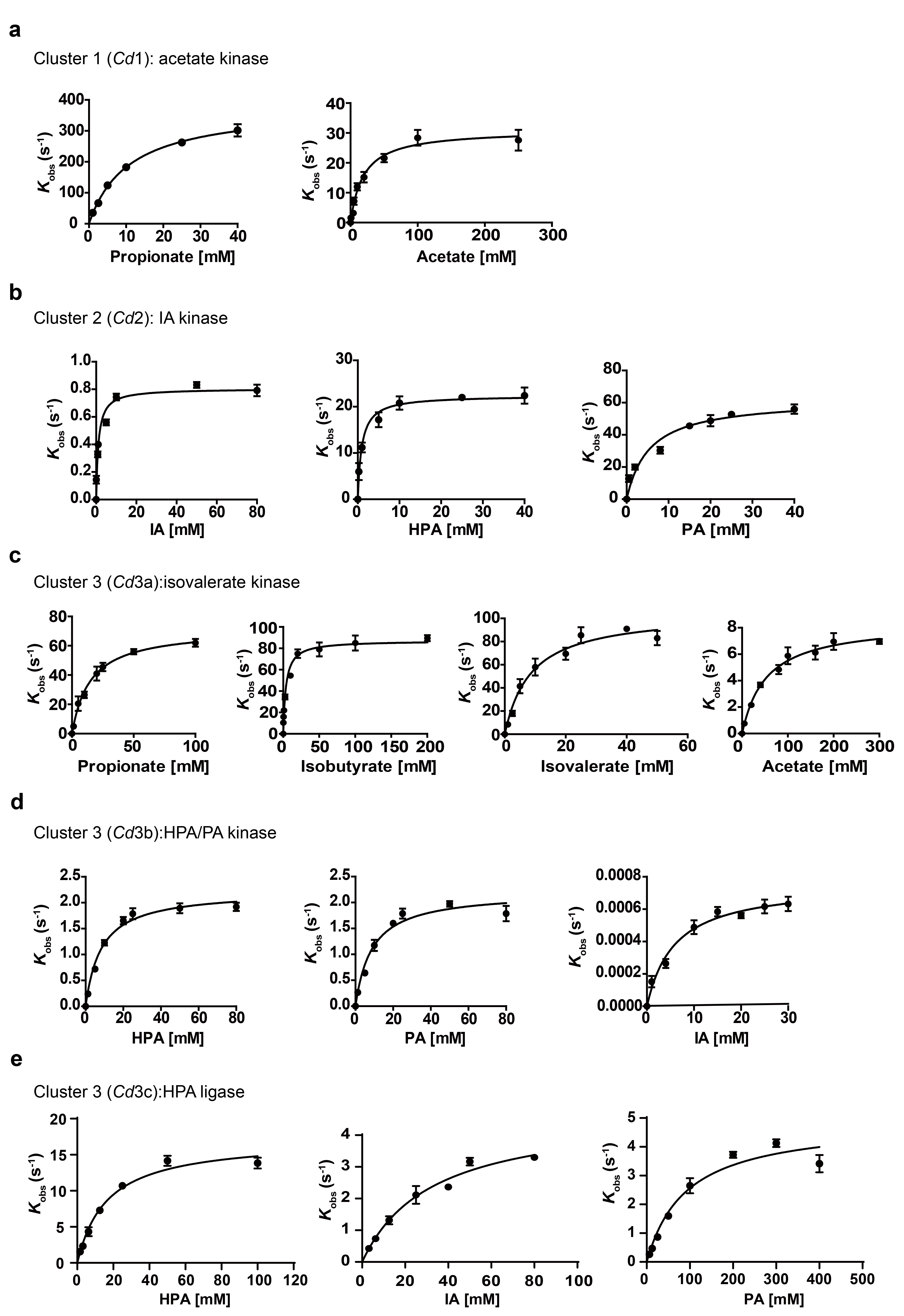


**Figure S10. Kinetic assays on selected kinases and ligase using different substrates.** ADP formation was coupled by PK and LDH to the decrease of NADH. The reaction rate was monitored by the decrease of absorbance at 340 nm. **a.** cluster 1 (*Cd*1). **b**. cluster 2 (*Cd*2). **c.** cluster 3 (*Cd*3a). **d.** cluster 3 (*Cd*3b) and **e.** cluster 3 (*Cd*3c) were analyzed for acetate, IA, isovalerate, HPA/PA kinases, and HPA ligase activity, respectively.


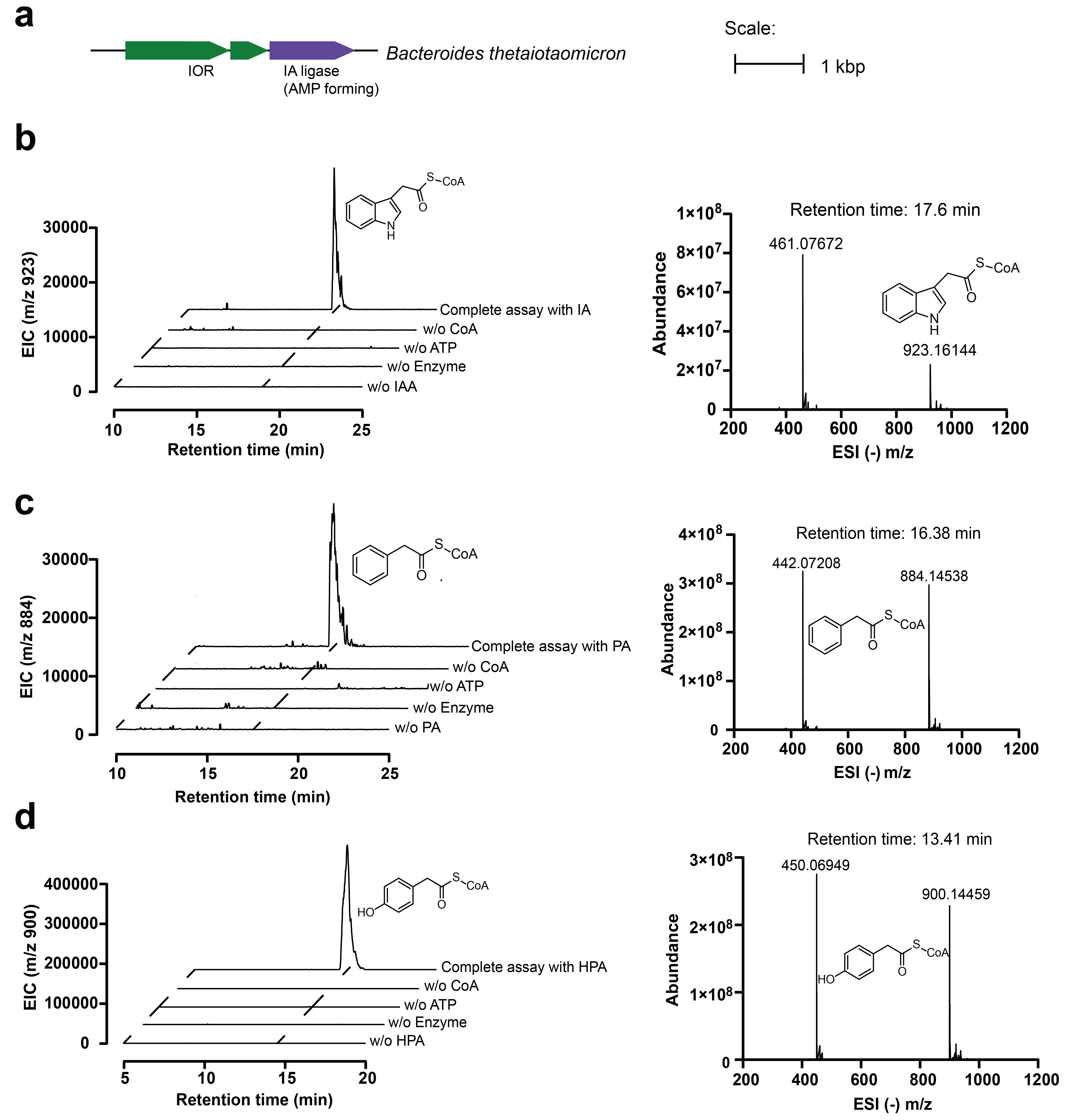


**Figure S11.** **Gene cluster of IA ligase and its enzymatic activity assay by HRLC-MS**. **a.** Gene cluster of IA ligase in *Bacteroides thetaiotaomicron*. **b-d.** Extracted ion chromatographs monitoring the formation of IA-CoA, PA-CoA and HPA-CoA, negative controls, and the ESI (-) m/z spectrum are presented, respectively.


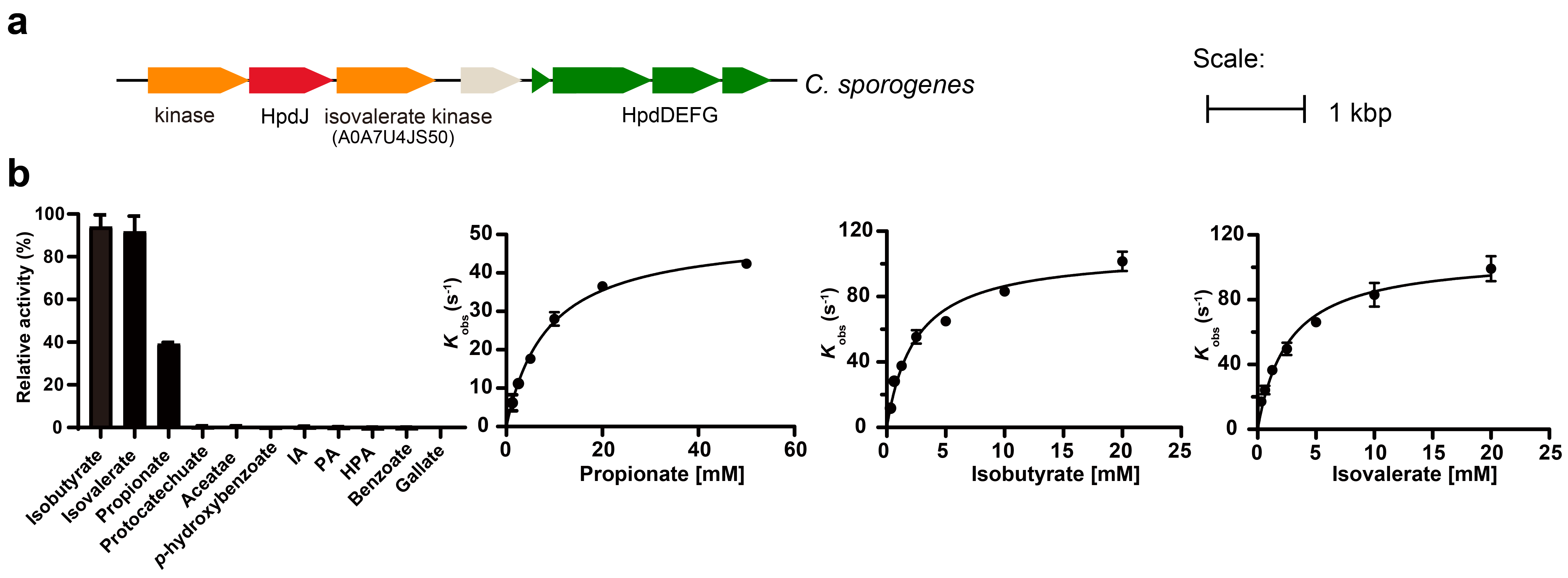


**Figure S12. Gene cluster and kinetics of isovalerate kinases. a.** Gene cluster of isovalerate kinase from *C. sporogenes*. **b.** Kinetic analysis of isovalerate kinase using different substrates.

**
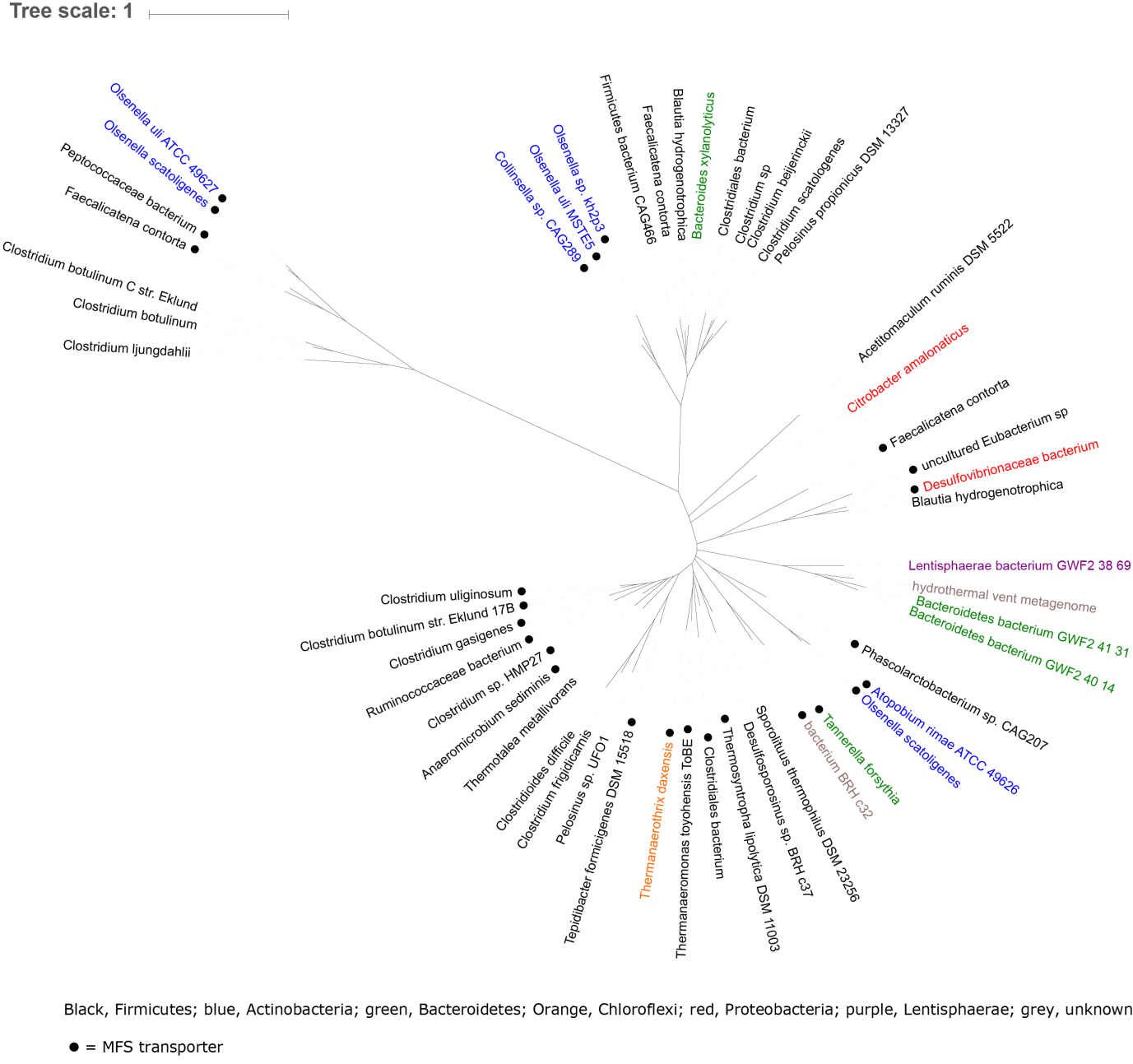
**

**Figure S13. Phylogenetic tree of candidate HPAD in different bacteria.** Black, Firmicutes; blue, Actinobacteria; green, Bacteroidetes; orange, Chloroflexi; red, Proteobacteria; purple, Lentisphaerae; grey, unknown. Black circle indicates the presence of a protein in the MFS transporter family (PF07690) within a 10 ORF window of HPAD.
